# Single nucleotide polymorphisms and haplotypes associated with feed efficiency in beef cattle

**DOI:** 10.1186/1471-2156-14-94

**Published:** 2013-09-25

**Authors:** Nick VL Serão, Dianelys González-Peña, Jonathan E Beever, Dan B Faulkner, Bruce R Southey, Sandra L Rodriguez-Zas

**Affiliations:** 1 Department of Animal Sciences, University of Illinois at Urbana-Champaign, Urbana, IL, USA; 2 Animal Sciences Department, University of Arizona, Tucson, AZ, USA; 3 Department of Statistics, University of Illinois at Urbana-Champaign, Champaign, IL, USA; 4 Institute for Genomic Biology, University of Illinois at Urbana-Champaign, Urbana, IL, USA

**Keywords:** Feed efficiency, Beef cattle, Single nucleotide polymorphism, Haplotype, Functional analysis, MAPK pathway, Serine/Threonine kinase activity

## Abstract

**Background:**

General, breed- and diet-dependent associations between feed efficiency in beef cattle and single nucleotide polymorphisms (SNPs) or haplotypes were identified on a population of 1321 steers using a 50 K SNP panel. Genomic associations with traditional two-step indicators of feed efficiency – residual feed intake (RFI), residual average daily gain (RADG), and residual intake gain (RIG) – were compared to associations with two complementary one-step indicators of feed efficiency: efficiency of intake (EI) and efficiency of gain (EG). Associations uncovered in a training data set were evaluated on independent validation data set. A multi-SNP model was developed to predict feed efficiency. Functional analysis of genes harboring SNPs significantly associated with feed efficiency and network visualization aided in the interpretation of the results.

**Results:**

For the five feed efficiency indicators, the numbers of general, breed-dependent, and diet-dependent associations with SNPs (P-value < 0.0001) were 31, 40, and 25, and with haplotypes were six, ten, and nine, respectively. Of these, 20 SNP and six haplotype associations overlapped between RFI and EI, and five SNP and one haplotype associations overlapped between RADG and EG. This result confirms the complementary value of the one and two-step indicators. The multi-SNP models included 89 SNPs and offered a precise prediction of the five feed efficiency indicators. The associations of 17 SNPs and 7 haplotypes with feed efficiency were confirmed on the validation data set. Nine clusters of Gene Ontology and KEGG pathway categories (mean P-value < 0.001) including, 9nucleotide binding; ion transport, phosphorous metabolic process, and the MAPK signaling pathway were overrepresented among the genes harboring the SNPs associated with feed efficiency.

**Conclusions:**

The general SNP associations suggest that a single panel of genomic variants can be used regardless of breed and diet. The breed- and diet-dependent associations between SNPs and feed efficiency suggest that further refinement of variant panels require the consideration of the breed and management practices. The unique genomic variants associated with the one- and two-step indicators suggest that both types of indicators offer complementary description of feed efficiency that can be exploited for genome-enabled selection purposes.

## Background

Feed efficiency is based on the relation between animal intake (input) and production (output). In beef cattle feedlot enterprises, the feed costs may represent up to 84% of the total costs, depending on the stage of production [1], and thus, improvement of feed efficiency may result in decreased production costs. Several indicators that address in different ways the complexities of feed efficiency have been proposed [2-7]. These indicators have distinct characteristics, such as correlation (overlap) with growth traits [8], absence of partition between the energy used for maintenance and production [2], use of ratios between variables with different variances [9], and failure to consider the level of uncertainty of estimates [2].

Several genome-wide association studies (GWAS) have identified single nucleotide polymorphisms (SNP) associated with feed efficiency in beef cattle [10-16]. The out breeding nature of beef cattle populations leads to differences in linkage disequilibrium (LD) between the alleles at SNP and at the loci controlling the trait, between breeds and between families within breeds. Thus, the association between SNPs and phenotypes may vary across breeds [10,13-16]. Similarly to breed-dependent associations, environment-dependent associations between SNPs and phenotypes, such as diet-by-SNP interactions, must be considered in GWAS studies [17,18].

In addition to individual SNP, the study of haplotype associations in GWAS has benefits. Haplotype blocks may capture epistatic interactions between SNPs [19-21], and drastically reduce the number of tests (control of type I error). The benefits of haplotype-based GWAS depend on the extent of LD between variants in the block and thus the length of the haplotype block and distance between the variants [22]. Few studies have reported associations between haplotypes and feed efficiency in beef cattle [23]. The study of haplotypes, rather than single SNP associations, has been proposed. The rationale are that haplotype provide more information to estimate whether two alleles are identical by descent, reduce the number of tests and hence the type I error rate, allow informed testing between clades of haplotype alleles by capturing information from evolutionary history, and provide more power than single SNPs when an allelic series exists at a locus [21]. However, these arguments have caveats [21]. The information content of haplotypes is dependent on the particular mutational and recombinational history of the loci and nearby SNPs. Also, the distribution of loci and SNP variants are not parallel across the genome, and thus haplotype information could capture associations that would elude single SNPs [21]. Following recommendations, both single SNP and haplotypes are investigated to take advantage of the full information content of the genotype data.

The objectives of this study were: 1) to identify general, breed- and diet-dependent associations between feed efficiency in beef cattle and SNPs or haplotypes and, 2) to compare the genomic associations with traditional two-step indicators of feed efficiency – residual feed intake (RFI), residual average daily gain (RADG), and residual intake gain (RIG) – relative to two complementary one-step models of feed efficiency: efficiency of intake (EI) and efficiency of gain (EG). Associations uncovered in a training data set were evaluated on a validation data set. A multi-SNP model was developed to predict feed efficiency. Functional analysis and network visualization aided in the interpretation of the results.

## Methods

### Animals and data description

Animals used in this trial were managed according to the guidelines recommended in the Guide for the Care and Use of Agricultural Animals in Agricultural Research and Teaching [24]. Experimental protocols were submitted to and approved by the University of Illinois Institutional Animal Care and Use Committee [25].

A beef cattle population encompassing various breed compositions and receiving various diets was used to uncover general and breed- or diet-specific genomic variants associated with feed efficiency. A total of 1,321 feedlot steers obtained from five ranches in Montana representing calving years between 2005 and 2008 were studied. Steers entered the study with an average (± standard deviation) of 249.75 ± 29.91 days of age, and stayed for an average (± standard deviation) test period of 160.96 ± 16.50, 180.08 ± 15.20, 163.39 ± 13.31, and 158.68 ± 14.78 days, for years 2005 to 2008, respectively. Animals were harvested in different groups, from two to three groups per year and ranch. All ranches participated in this study during all years and one ranch was used only in the last year. The combination of these harvest groups, ranches, and years was used to create contemporary groups (CG, 27 levels). The pedigree and breed information of all steers used in this study were accessed from the American Simmental Association Herdbook Service [26]. The pedigree included a total of 3,331 animals. After individual verification, steers pertaining to one of five breed compositions: purebred Angus (AN), 3/4 Angus (3/4AN), crossbred Angus and Simmental (ANSM), 3/4 Simmental (3/4SM) and purebred Simmental (SM). All breed compositions were represented across all years and harvest groups, and 3/4AN and AN were present in four ranches. The steers received one of 12 diets in which many showed similar composition and nutritional value. Thus, the diets were further grouped into five levels according to the main ingredient, total net energy, and non-degradable fiber as shown in Table 1. All five diets were represented across all breed and harvest group levels. Diets C and E were not used in one of the ranches, and diets A, B and D were represented in all years.

**Table 1:** **Description of the diets**^**1**^

**Item**	**Dietary treatment**				
	**A**	**B**	**C**	**D**	**E**
TNE, Mcal/lb	1.40	1.15	1.15	1.15	1.09
NDF,%	18.5	39.2	41.5	40.1	45.1
DM,%	66.7	63	65	54	49
CP,%	13.9	18.8	14.4	17.7	21.4
ADF,%	7.8	21.9	23.6	22.8	25.6
TDN,%	75.7	67.5	68	68	66
Main ingredients	Dry-rolled corn and stored wet distiller grain	Distiller grains with solubles and fresh wet corn gluten feed	Dry-rolled corn and corn gluten feed	Fresh wet distiller grains and wet corn gluten feed	Stored wet distiller grains and hay

^1^Average values for each item across all diets used to define each dietary treatment.

*TNE* Total net energy, *NDF* Non-degradable fiber, *DM* Dry matter, *CP* Crude protein, *ADF* Acid detergent fiber, *TDN* Total digestible nutrient.

After one week of adaptation to the diet, initial weight (IW, kg) of each animal was recorded and steers were measured for dry matter intake (DMI, kg/day), rib eye area (REA, cm^2^), and back fat thickness (BF, cm). Adjusted final weight (FW, kg) was obtained as hot carcass weight divided by the average dressing percentage of the harvest group, and used to calculate individual average daily gain (ADG, kg), as the difference between FW and IW, divided by the test period. The calculation of ADG in this study may not be optimal since the FW was estimated using the average dressing percentage of the harvest group. Individual feed intake records were collected using the GrowSafe automated feeding system (GrowSafe Systems Ltd., Airdrie, Alberta, Canada). Chromatography paper was used to take an image of the *longissimus dorsi* for REA measures, and recorded using a planometer. Measures of BF were taken in a transverse orientation between the 12th and 13th ribs, at approximately 10 cm distal from the midline. The average (± standard deviation) of IW, ADG, DMI, REA, and BF were 310.10 ± 40.08 kg, 1.61 ± 0.24 kg, 10.48 ± 1.42 kg/day, 90.18 ± 10.19 cm^2^, and 1.26 ± 0.36 cm, respectively. A more detailed description of the diets, measurements of the traits, and slaughter procedures may be found elsewhere [25].

### Feed efficiency indicators

Established and new indicators of feed efficiency were evaluated. Among the known indicators, RFI was calculated as the difference between the observed and predicted DMI [2]. The DMI values were predicted using a linear model including ADG, mid-test metabolic body weight (MBW; mid-test BW^0.73^), REA, and BF. In a similar fashion, RADG (also known as residual body weight gain; RG [6]) was calculated subtracting the observed ADG from its prediction. The ADG values were predicted using a linear model including DMI, MBW, REA, and BF. Residual intake and gain (RIG) was calculated as the difference between RADG and RFI, and both are standardized to unit variance [7]. Positive values for RADG and RIG, and negative values for RFI, are indicators of higher feed efficiency. Predictions of DMI and ADG, as well as computation of RFI, RADG, and RIG, were performed using the MIXED procedure is SAS 9.2 (Statistical Analysis System Institute, Inc., Cary, NC, USA). A summary of the models used to predict ADG and DMI is presented in Table 2.

**Table 2:** Overall performance of the models used to predict ADG and DMI

	**P-value**^**1**^					
Phenotype	MBW	REA	BF	ADG	DMI	r^2^
ADG	<0.0001	<0.0001	0.0006	-	<0.0001	39.49%
DMI	<0.0001	<0.0001	0.0327	<0.0001	-	42.75%

^1^*MBW* metabolic body weight (mid-test BW^0.73^), *REA* rib eye area, *BF* back fat thickness, *ADG* average daily gain, *DMI* dry matter intake.

The identification of SNPs associated with RADG or RFI is the result of a two-step approach. The first step consists of the estimation of ADG (or DMI) once the covariation due to MBW, DMI (or ADG), REA, and BF have been removed. The second step consists on the identification of SNPs or haplotypes associated with the resulting point residuals of ADG (or DMI). These residuals are a function of point predictions that ignore the uncertainty (e.g. confidence intervals) associated with these predictions. The purpose of these calculations of RFI, RADG, and RIG is to minimize the effects of body weight [7]. A one-step SNP association approach can achieve comparable independence while accommodating the uncertainty of the predictions. In order to differentiate RFI and RADG from the proposed methods that follow, these will be called ‘two-step indicators’ for the rest of the paper.

Two complementary indicators of feed efficiency obtained from a one-step model for SNP associations are proposed. In the one-step model, the association between SNPs or haplotypes and feed efficiency is described in one model that includes the covariates used in the first step of the computation of RFI and RADG. The complementary one-step model overcomes the limitation of RFI and RADG by accommodating parameter estimate uncertainty. The one-step indicators used as complements for RFI and RADG are termed efficiency of intake (EI) and efficiency of gain (EG), respectively.

### Genotyping and quality control

Genotypes were obtained using the Illumina® BovineSNP50 BeadChips v1 and v2 platforms (Illumina Inc., San Diego, CA) that include 54,001 and 54,609 SNPs, respectively. The 52,340 SNPs presented in both versions of the platform were analyzed. Quality control was performed in two steps. First, SNPs not assigned to chromosomes, according to the Bos_taurus_UMD_3.1 assembly (519 SNPs) [27], and with GenCall scores below 0.2 (16 SNPs) were excluded from further analyses. GenCall scores below 0.2 generally indicate failed genotypes [28]. In the second step, quality control was implemented using PLINK [29]. Observations were removed when not meeting the following thresholds: steer missingness per SNP (< 20%) [30], Hardy-Weinberg equilibrium test (P-value > 0.00001) [31], SNP missingness per steer (< 10%) [32], and minor allele frequency (MAF > 5%) [32]. Respectively, 264 SNPs, 1,202 SNPs, 9 steers, and 9,811 SNPs were not considered for further analysis. The final data set included 1,312 steers and 40,528 SNPs, with a total genotyping rate of 99.55%.

### Haplotype reconstruction

Using the genotypic data after quality control, haplotype blocks were and phased using PLINK. Blocks were estimated within a 200 kb window [33] using the confidence interval method [34], resulting in 1,129 non-overlapping haplotype blocks. The size of the window offers a compromise between encompassing SNPs in LD while controlling for the number of alleles. A large number of alleles could jeopardize the representation of alleles across breeds and diets and could result in spurious significant contrasts between pairs of alleles. In cattle, linkage disequilibrium does not extend substantially beyond 500 kb (r^2^ < 0.08) and is low at 100 kb (r^2^ < 0.18) [33,35]. A window of 200 kb was selected to generate blocks that will encompass most LD and the average number of alleles was 3.88 per block. The average number of SNPs per block was 3.38 and ranged from two to seven. The average block size was 91.38 kb and ranged from 0.001 kb to 199.644 kb.

The association between feed efficiency and non-overlapping blocks was tested. The lower number of non-overlapping blocks, relative to sliding window overlapping blocks, resulted in a substantial reduction in the number of tests and thus in less stringent multiple-test adjustment of the significance P-values. A potential drawback of the non-overlapping approach is that all SNPs in LD with the loci could be assigned to different blocks, thus resulting in loss of power. This situation may also arise for the sliding window approach when a limited number of window widths are applied [36]. In this case, the single SNP analysis in the present study will detect numerous nearby SNPs associated in various degrees with feed efficiency.

Haplotype alleles with posterior phasing probability below 1 were excluded to increase the reliability of the haplotype alleles. The second quality control step in the haplotype data used the same thresholds from the individual SNP analysis, and no blocks or steers were removed. The extent of LD between SNPs within haplotype blocks was computed in PLINK using the pairwise average *r*^*2*^ statistics [37] in order to assess the recombination potential in these genomic regions.

### Training and validation data set

Two data sets were obtained from the records that passed the quality control. The training data set consisted of 976 steers (75% of the total number of steers) and was used to identify SNPs and haplotypes associated with feed efficiency. The validation data set consisted of 336 steers (25% of the total number of steers) and was used to validate the findings. Steers from the same sire were assigned to only one of the two data sets to minimize the dependencies between data sets and attain a less biased validation [15,38]. The number and proportion of steers on each data set, by the levels of diet and breed, are presented in Table 3.

**Table 3:** Number (proportion) of steers by breed and diet within data sets

**Breed**	**Training ( *n = 976 *)**	**Validation ( *n = 336 *)**
AN	102 (0.10)	35 (0.10)
3/4AN	115 (0.12)	67 (0.20)
ANSM	640 (0.66)	190 (0.57)
3/4SM	39 (0.04)	19 (0.06)
SM	80 (0.08)	25 (0.07)
Diet		
A	232 (0.24)	83 (0.25)
B	300 (0.31)	88 (0.26)
C	111 (0.11)	48 (0.14)
D	257 (0.26)	105 (0.31)
E	76 (0.08)	25 (0.07)

### Statistical analyses

#### Whole-genome SNP and haplotype association analysis

The general model used to identify associations between SNPs and the two-step feed efficiency indicators RFI, RADG, and RIG was:

(1) $$\begin{array}{l} Y_{\mathrm{ijklm}}=\mu +\mathrm{SN}P_{i}+B_{j}+D_{k}+{\left(\mathrm{SNP}*B\right)}_{\mathrm{ij}}\\ \;+{\left(\mathrm{SNP}*D\right)}_{\mathrm{ik}}+CG_{l}+b_{1}\left(IW_{\mathrm{ijklm}}-\bar{\mathrm{IW}}\right)\\ \;+a_{\mathrm{ijklm}}+e_{\mathrm{ijklm}} \end{array}$$

where Y_ijklm_ is the value of RFI, RADG, or RIG, μ is the overall mean, SNP_i_ is the fixed effect of the SNP genotype, B_j_ is the fixed effect of breed (5 levels), D_k_ is the fixed effect of diet (5 levels), CG_l_ is the random effect of contemporary group (27 levels, [0, $\sigma_{\mathrm{CG}}^{2}$ ]), b_1_ is the fixed effect regression coefficient for the covariate IW, a_ijklm_ is the random animal effect (0, $\;A\sigma_{a}^{2}$; where **A** is the additive relationship matrix), and e_ijklm_ is the random error (0, $\;\sigma_{e}^{2}$) associated with Y_ijklm_. The analysis included the covariate IW due to its correlation with DMI and ADG [5]. In addition, preliminary analysis were undertaken in order to compare the use of IW and initial age in the model, and lower root mean error (RMSE) was obtained for the model including IW.

For the complementary one-step models, Eq. 1 was extended to include the adjustments previously detailed for RFI and RADG, resulting in the following models for, respectively, EI (Eq. 2) and EG (Eq. 3):

(2) $$\begin{array}{l} \mathrm{DM}I_{\mathrm{ijklm}}=\mathrm{Eq}.1+b_{2}\left(\mathrm{AD}G_{\mathrm{ijklm}}-\bar{\mathrm{ADG}}\right)\\ \;+b_{3}\left(\mathrm{MB}W_{\mathrm{ijklm}}-\bar{\mathrm{MBW}}\right)\\ \;+b_{4}\left(BF_{\mathrm{ijklm}}-\bar{\mathrm{BF}}\right)\\ \;+b_{5}\left(\mathrm{RE}A_{\mathrm{ijklm}}-\bar{\mathrm{REA}}\right) \end{array}$$

(3) $$\begin{array}{l} \mathrm{AD}G_{\mathrm{ijklm}}=\mathrm{Eq}.1+b_{6}\left(\mathrm{DM}I_{\mathrm{ijklm}}-\bar{\mathrm{DMI}}\right)\\ \;+b_{7}\left(\mathrm{MB}W_{\mathrm{ijklm}}-\bar{\mathrm{MBW}}\right)\\ \;+b_{8}\left(BF_{\mathrm{ijklm}}-\bar{\mathrm{BF}}\right)\\ \;+b_{9}\left(\mathrm{RE}A_{\mathrm{ijklm}}-\bar{\mathrm{REA}}\right) \end{array}$$

where b_2_, b_3_, b_4_, and b_5_ are the fixed effect regression coefficients for the covariates ADG, MBW, BF, and REA, respectively, for the EI model, and b_6_, b_7_, b_8_, and b_9_ are the fixed effect regression coefficients for the covariates DMI, MBW, BF, and REA, respectively, for the EG model. The additive and dominance effects of the SNPs were tested jointly and SNPs were considered significant at P-value < 0.0001 [16].

Similar models were used to test the individual association between the 1,129 haplotype blocks and the five indicators by substituting SNP for haplotype in the models. The additive effect of haplotype was tested and considered significant at P-value < 0.001 to account for the lower number of haplotype hypotheses tested (1,129 haplotypes) compared to SNP hypotheses tested (40,528 SNPs).

Additive and dominance estimates are presented relative to the less frequent or minor allele. The additive effect was tested for SNPs located on BTA X, when testing the interactions with breed and with diet, and for the haplotype analyses. The additive estimates for SNPs interacting with breed or diet are the contrasts between each breed level relative to SM steers for breed-by-SNP associations, and each diet level relative to diet E, for diet-by-SNP associations. Before association analyses, the normality and homoscedastic of the residual estimates from each model without the SNP effect was confirmed assessing the Shapiro-Wilk’s test of normality using the UNIVARIATE procedure in SAS. All association analyses were performed using Qxpak v.5.05 [39].

#### Multi-SNP model selection

A SNP-based indicator of feed efficiency was developed by simultaneously considering significant SNPs and their ability to predict each of the five feed efficiency indicators evaluated using a stepwise selection approach. All the explanatory variables in Equations 1, 2, and 3 were kept in the model with the exception of the SNPs that underwent variable selection. For each feed efficiency indicator, the set of SNPs that were significantly associated at P-value < 0.001 in the whole-genome SNP association analysis were assessed for entering and staying in the model at P-value < 0.0001.

#### SNP and haplotype validation

The significant SNPs and haplotypes identified in the genome-wide analyses were evaluated in the validation data set using the same models. Both, the significance level and trend (sign of the estimates) of the SNP and haplotype were compared between the two independent data sets. Each SNP and haplotype was individually tested and considered validated at P-value < 0.05 [14]. For the multi-SNP models, the model adequacy (MA; Eq. 4) was assessed by comparing the estimated RMSE between the training and the validation data sets using the following formula:

(4) $$\mathrm{MA}=\left(1-\frac{\mathrm{RMS}E_{T}}{\mathrm{RMS}E_{V}}\right)*100\%$$

where RMSE_T_ and RMSE_V_ are the root mean square errors of the models when using the training and validation data sets, respectively. In this formula it is possible to assess the drop in the model adequacy when fitting the set of selected SNPs using the validation data set in place of the training data set.

#### Genetic parameter

Heritability estimates were obtained using single trait analyses, whereas the genetic and phenotypic correlations were obtained using bivariate analyses. The five feed efficiency indicators were analyzed using the models described for whole-genome analysis, excluding SNP or haplotype and the interactions with other model factors. The variance components of the indicators were estimated using WOMBAT [40].

#### Functional and gene network analyses

The Gene Ontology (GO) categories that are overrepresented among the genes harboring the SNPs associated with feed efficiency were identified. This analysis and gene network visualization offered insights into molecular functions and biological processes that could be associated with feed efficiency in beef cattle. Gene Ontology [41] categories and KEGG pathways [42] that were enriched among the genes harboring SNPs and intergenic SNPs (SNPs within 2 kb 5′ or 0.5 kb 3′ to a gene [28]) associated with feed efficiency at P-value < 0.01 were identified using the functional annotation clustering option in DAVID [43]. Genes more distant from the detected SNPs were not included in the functional analysis because the potential number of spurious genes added to the functional analysis could overwhelm the fewer potential true loci, thus biasing the results. Enrichment of FAT categories that include molecular function and biological process was investigated. Gene Ontology FAT categories are a subset of the broadest GO terms, which are filtered so that they do not overshadow the more specific terms.

Enrichment was identified in three gene lists: general SNP associations, breed-by-SNP associations, and diet-by-SNP associations that resulted from the combination of all five feed efficiency indicators. In order to identify background genes for the enrichment, the SNP-encompassing sequences (provided by Illumina Inc., San Diego, CA) were mapped to the Bos Taurus genome assembly UMD_3.1 using BLASTN. Only annotated genes that contained the best hit of each SNP with an E-value < 1.0E-10 were retained as background genes for enrichment testing. An EASE score was applied to evaluate each functional category using the Fisher Exact test [43]. The EASE score was calculated by removing one gene from the list of significant genes within the tested functional category [43]. Functional annotation clustering was used to reduce redundancies from similar annotations repeatedly listed among the results. Categories that shared genes were grouped together in a cluster to facilitate interpretation. In addition to the P-values of the individual categories, a group enrichment score of the categories in a cluster (the geometric mean of the category P-values in –log scale) was computed. Functional annotation clusters with enrichment score > 3 (P-value geometric mean < 1.0E-03) were considered significant and reported.

Gene networks associated with feed efficiency were inferred based on the lists of genes corresponding to the significant GO terms. Networks were visualized using the BisoGenet plug-in [44] from the Cytoscape software [45]. All the available data sources in BisoGenet (including BIOGRID, DIP, BIND and others) were selected to generate the interactions, in which are represented by the edges between two genes (nodes). The final pathway included genes separated by at most one intermediate gene to highlight the interactions between the target genes. Target genes are represented by pink nodes, while intermediate genes are by blue nodes. The size of the network nodes from the target genes is a function of the P-values from the association analyses, in which larger nodes indicate more significant P-values, while the size of the nodes of intermediate genes are constant. The node size of genes with more than one SNP associated was represented as the average P-values of the SNPs. Nodes with self-edges represent genes presenting self-regulation function.

## Results and discussion

### General results

A summary of the total number of unique significant SNPs (P-value < 0.0001) and haplotypes (P-value < 0.001) are presented in Tables 4 and 5, respectively. In the SNP and haplotype analyses, RIG showed the highest number of unique associations, followed by the intake-based indicators RFI and EI, and lastly the gain-based indicators RADG and EG. The traditional indicators RFI and RADG had a similar number of associations than the respective complementary indicators EI and EG in both the analyses. Of the 21 SNPs associated with RFI and any other indicator, 18 were also associated with EI, and in all cases the same type of association (general or breed/diet-dependent) was significant. Similarly, of the 7 SNPs associated with RADG and any other indicator, 5 were also associated with EG, always with the same type of association. The overlap between the RFI and RADG associations and the EI and EG associations, suggests that the latter indicators may have the potential to be used in lieu of the former ones, respectively.

**Table 4:** **Number of SNPs significantly associated**^**1 **^**with feed efficiency and genes harboring SNPs by association and indicator**

	**Type of SNP association**							
**Indicator**	**General**		**Breed-dependent**		**Diet-dependent**		**Total**^**2**^	
	**SNPs**	**Genes**	**SNPs**	**Genes**	**SNPs**	**Genes**	**SNPs**	**Genes**
RFI	7	2	10	4	12	2	26	9
RADG	9	3	9	5	1	1	19	6
RIG	8	1	20	6	9	2	37	9
EI	10	3	8	5	16	4	31	10
EG	13	8	4	2	1	1	18	8
Total^2^	31	11	40	15	25	6	93	29

^1^P-value < 0.0001.

^2^Total number of unique SNPs and genes.

**Table 5:** **Number of haplotypes significantly associated**^**1 **^**with feed efficiency and genes harboring SNPs by association and indicator**

	**Type of SNP association**							
**Indicator**	**General**		**Breed-dependent**		**Diet-dependent**		**Total**^**2**^	
	**Haplotypes**	**Genes**	**Haplotypes**	**Genes**	**Haplotypes**	**Genes**	**Haplotypes**	**Genes**
RFI	4	6	2	2	2	2	8	10
RADG	1	0	4	6	1	0	6	6
RIG	1	1	6	8	4	4	11	13
EI	2	2	2	2	3	2	7	6
EG	0	-	0	-	1	0	1	0
Total^2^	6	6	10	12	9	4	20	22

^1^P-value < 0.0001.

^2^Total number of unique haplotypes and genes.

### Genetic parameters of feed efficiency

The heritability estimates for RFI, RADG, RIG, EI, and EG were: 0.40 ±0.10, 0.17 ± 0.07, 0.40 ± 0.10, 0.40 ± 0.10, 0.16 ± 0.07, respectively. Consistent with SNP and haplotype results, the heritability for RFI and RADG are similar to EI and EG, respectively. The RFI heritability estimate is consistent with those previously reported [4,7,8]. The RADG heritability estimate is slightly lower than that (0.28) reported in another study [6]. Similarly, our results are comparable to the heritability estimate for RIG in beef cattle (0.36) previously reported [7].

All absolute genetic and phenotypic correlation estimates, were above 0.9 with the exception of RADG with RFI and RIG. The high correlations are expected considering the similarity between indicators. The genetic and phenotypic correlation estimates between RADG and RFI were, 0.43 ± 0.09 and −0.34 ±0.03, respectively, and are in accordance with previous reports [6]. The genetic and phenotypic correlation estimates between RADG and RIG were 0.55 ± 0.08 and 0.48 ± 0.03, respectively, and agree in sign with previous studies (0.83 and 0.85, respectively) [7]. The similarity of the genetic parameter estimates between the two-step indicators RFI and RADG, and the one-step indicators EI and EG, respectively, further support the proposition that the complementary indicators can be used as proxy for the traditional counterparts.

### Whole-genome SNP association analysis

The SNPs significantly associated (P-value < 0.0001) with each indicator *per se* or interacting with breed or diet that were mapped to gene regions are presented in Tables 6, 7, and 8, for respectively. The complete list including SNPs not mapped to genes is provided in the Additional file 1: Tables S1, S2, and S3, for the respective associations.

**Table 6:** **Additive**^**1 **^**and dominance estimates of SNPs within genes that have general association**^**2 **^**with feed efficiency**

**Indicator**	**SNP**	**BTA**	**Allele**	**Gene symbol**	**Gene name**	**Additive**^**3**^	**Dominance**^**3**^	**P-Value**
RFI	rs109500421	8	C/T^*^	*CNTFR*	Ciliary neurotrophic factor receptor	−0.01 ± 0.05	0.24 ± 0.06	4.54E-05
	rs108942504	22	A/G^*^	*TMEM40*	Transmembrane protein 40	0.36 ± 0.10	−0.09 ± 0.11	9.04E-06
RADG	rs108964818	15	C/T^*^	*KDELC2*	KDEL (Lys-Asp-Glu-Leu) containing 1-like	0.35 ± 0.06	0.34 ± 0.06	3.44E-08
	rs41620774	15	A/C^*^	*ELMOD1*	ELMO/CED-12 domain containing 1	0.12 ± 0.03	0.13 ± 0.03	5.58E-05
	rs42342964	23	G^*^/T	*PAK1IP1*	PAK1 interacting protein 1	0.01 ± 0.01	0.05 ± 0.01	9.16E-06
RIG	rs108964818	15	C/T^*^	*KDELC2*	KDEL (Lys-Asp-Glu-Leu) containing 1-like	2.96 ± 0.56	2.83 ± 0.57	7.43E-07
EI	rs109500421	8	C/T^*^	*CNTFR*	Ciliary neurotrophic factor receptor	−0.02 ± 0.05	0.23 ± 0.06	5.03E-05
	rs109709275	15	A/G^*^	*GRAMD1B*	GRAM domain containing 1B	0.05 ± 0.05	−0.18 ± 0.05	6.29E-05
	rs108942504	22	A/G^*^	*TMEM40*	Transmembrane protein 40	0.32 ± 0.10	−0.05 ± 0.11	2.29E-05
EG	rs110340232	1	G^*^/T	*RAB6B*	RAB6B, member RAS oncogene family	0.01 ± 0.01	0.04 ± 0.01	5.07E-05
	rs110787048	4	A^*^/G	*DPP6*	Dipeptidyl-peptidase 6	−0.03 ± 0.01	−0.04 ± 0.01	9.32E-05
	rs110051312	8	A^*^/C	*PTPN3*	Protein tyrosine phosphatase, non-receptor type 3	0.04 ± 0.03	0.09 ± 0.03	5.46E-05
	rs110196238	8	C^*^/T	*PTPN3*	Protein tyrosine phosphatase, non-receptor type 3	0.04 ± 0.03	0.09 ± 0.03	5.09E-05
	rs41611457	12	A/G^*^	*ENOX1*	Ecto-NOX disulfide-thiol exchanger 1	−0.04 ± 0.01	0.03 ± 0.01	6.70E-05
	rs108964818	15	C/T^*^	*KDELC2*	KDEL (Lys-Asp-Glu-Leu) containing 1-like	0.37 ± 0.07	0.37 ± 0.07	4.42E-07
	rs41620774	15	A/C^*^	*ELMOD1*	ELMO/CED-12 domain containing 1	0.12 ± 0.03	0.15 ± 0.03	2.94E-05
	rs109889052	19	C/T^*^	*PIK3R6*	Phosphoinositide-3-kinase, regulatory subunit 6	−0.27 ± 0.07	0.29 ± 0.07	7.79E-05

^1^Additive estimate relative to the minor allele.

^2^P-value < 0.0001;

^3^Estimate ± standard error;

^*^Minor allele.

**Table 7:** **Additive**^**1 **^**estimates of SNPs within genes that have breed-dependent association**^**2 **^**with feed efficiency**

**Indicator**	**SNP**	**BTA**	**Allele**	**Gene symbol**	**Gene name**	**Breed**^**3**^				**P-Value**
						**AN**	**3/4 AN**	**AN/SM**	**3/4 SM**	
RFI	rs110425294	5	A/G^*^	*AVIL*	Advillin	−0.09 ± 0.06	0.09 ± 0.06	0.13 ± 0.05	−0.23 ± 0.08	5.71E-05
	rs42456314	12	A/G^*^	*GPC5*	Glypican 5	−0.08 ± 0.07	−0.27 ± 0.07	−0.20 ± 0.06	−0.57 ± 0.08	2.69E-05
	rs29024448	17	G/T^*^	*RFC5*	Replication factor C (activator 1) 5, 36.5 kDa	0.14 ± 0.06	0.31 ± 0.06	0.05 ± 0.05	0.20 ± 0.07	8.32E-06
	rs108942504	22	A/G^*^	*TMEM40*	Transmembrane protein 40	0.05 ± 0.13	0.15 ± 0.12	0.17 ± 0.10	0.53 ± 0.13	3.07E-06
RADG	rs109808044	3	A^*^/G	*SNED1*	Sushi, nidogen and EGF-like domains 1	−0.04 ± 0.01	−0.08 ± 0.01	−0.01 ± 0.01	−0.03 ± 0.01	2.44E-05
	rs110742206	3	C/T^*^	*CSMD2*	CUB and Sushi multiple domains 2	0.11 ± 0.03	0.11 ± 0.03	0.08 ± 0.02	0.23 ± 0.04	1.98E-05
	rs110690110	5	C^*^/G	*ERC1*	ELKS/RAB6-interacting/CAST family member 1	−0.03 ± 0.01	−0.05 ± 0.01	−0.02 ± 0.01	−0.07 ± 0.01	2.49E-05
	rs110280556	6	A^*^/G	*UNC5C*	Unc-5 homolog C (C. elegans)	−0.02 ± 0.01	−0.03 ± 0.01	0.00 ± 0.01	0.05 ± 0.01	6.65E-06
	rs41583989	24	C^*^/T	*DTNA*	Dystrobrevin, alpha	−0.07 ± 0.02	0.04 ± 0.02	−0.02 ± 0.01	−0.04 ± 0.02	9.20E-05
RIG	rs110131536	2	A^*^/G	*IGFBP5*	Insulin-like growth factor binding protein 5	0.33 ± 0.14	0.40 ± 0.13	0.47 ± 0.12	−0.69 ± 0.20	2.30E-05
	rs110280556	6	A^*^/G	*UNC5C*	Unc-5 homolog C (C. elegans)	−0.13 ± 0.08	−0.11 ± 0.08	0.06 ± 0.06	0.62 ± 0.11	3.88E-05
	rs41625438	12	C/T^*^	*DACH1*	Dachshund homolog 1 (Drosophila)	0.06 ± 0.15	0.03 ± 0.15	0.05 ± 0.13	1.08 ± 0.21	8.82E-05
	rs42456314	12	A/G^*^	*GPC5*	Glypican 5	0.22 ± 0.10	0.41 ± 0.10	0.36 ± 0.08	0.90 ± 0.12	2.35E-06
	rs41623603	16	A^*^/C	*CNST*	Consortin, connexin sorting protein	0.08 ± 0.10	0.10 ± 0.11	0.19 ± 0.09	−0.36 ± 0.13	5.13E-05
	rs29024448	17	G^*^/T	*RFC5*	Replication factor C (activator 1) 5, 36.5 kDa	−0.15 ± 0.09	−0.48 ± 0.09	−0.09 ± 0.07	−0.32 ± 0.11	1.71E-05
EI	rs109053103	5	A^*^/G	*BIN2*	Bridging integrator 2-like	0.28 ± 0.08	0.28 ± 0.08	−0.01 ± 0.06	0.36 ± 0.10	8.57E-05
	rs42456314	12	A/G^*^	*GPC5*	Glypican 5	−0.10 ± 0.07	−0.26 ± 0.07	−0.21 ± 0.06	−0.55 ± 0.08	3.13E-05
	rs29024448	17	G^*^/T	*RFC5*	Replication factor C (activator 1) 5, 36.5 kDa	0.11 ± 0.06	0.30 ± 0.06	0.07 ± 0.05	0.19 ± 0.07	3.23E-05
	rs108942504	22	A/G^*^	*TMEM40*	Transmembrane protein 40	0.05 ± 0.12	0.09 ± 0.11	0.16 ± 0.10	0.48 ± 0.13	8.72E-06
	rs110206384	X	A/G^*^	*F8*	Coagulation factor VIII, procoagulant component	0.17 ± 0.05	−0.07 ± 0.05	−0.02 ± 0.04	0.01 ± 0.07	5.48E-05
EG	rs110742206	3	C/T^*^	*CSMD2*	CUB and Sushi multiple domains 2	0.54 ± 0.13	0.46 ± 0.12	0.35 ± 0.11	1.08 ± 0.17	7.31E-05
	rs42250803	17	A^*^/G	*SLC7A11*	Solute carrier family 7 (anionic amino acid transporter light chain, xc- system), member 11	0.06 ± 0.06	−0.06 ± 0.05	0.02 ± 0.04	−0.31 ± 0.06	2.23E-05

^1^Contrast of the additive estimate between each level of breed and breed SM, relative to the minor allele.

^2^P-value < 0.0001.

^3^Estimate ± standard error.

^*^Minor allele.

**Table 8:** **Additive**^**1 **^**estimates of SNPs within genes that have diet-dependent association**^**2 **^**with feed efficiency**

**Indicator**	**SNP**	**BTA**	**Allele**	**Gene symbol**	**Gene name**	**Diet**^**3**^				**P-Value**
						**A**	**B**	**C**	**D**	
RFI	rs108942504	22	A/G^*^	*TMEM40*	transmembrane protein 40	0.27 ± 0.08	0.28 ± 0.08	0.29 ± 0.09	0.22 ± 0.08	1.66E-05
RADG	rs43474365	7	A/G^*^	*SLC12A2*	solute carrier family 12 (sodium/potassium/chloride transporters), member 2	0.00 ± 0.01	−0.03 ± 0.01	0.01 ± 0.01	−0.03 ± 0.01	4.11E-05
RIG	rs108942504	22	A/G^*^	*TMEM40*	transmembrane protein 40	−0.51 ± 0.12	−0.40 ± 0.11	−0.46 ± 0.14	−0.32 ± 0.12	5.80E-05
EI	rs41593945	4	A^*^/C	*CNPY1*	canopy 1 homolog	−0.04 ± 0.07	−0.16 ± 0.07	0.22 ± 0.07	−0.02 ± 0.07	4.80E-05
	rs108942504	22	A/G^*^	*TMEM40*	transmembrane protein 40	0.21 ± 0.08	0.23 ± 0.07	0.24 ± 0.09	0.18 ± 0.08	8.28E-05
	rs42072585	25	A/G^*^	*CLN3*	ceroid-lipofuscinosis, neuronal 3	0.18 ± 0.06	0.21 ± 0.06	0.36 ± 0.06	0.27 ± 0.06	7.02E-05
EG	rs109291606	12	G/T^*^	*ENOX1*	ecto-NOX disulfide-thiol exchanger 1	0.04 ± 0.01	0.04 ± 0.01	0.04 ± 0.01	0.02 ± 0.01	8.93E-05

^1^Contrast of the additive estimate between each level of diet and diet E, relative to the minor allele.

^2^P-value < 0.0001.

^3^Estimate ± standard error.

^*^Minor allele.

Significant associations between feed efficiency and SNP-by-breed or SNP-by-diet interactions uncover breed- and diet-dependent SNP associations. The identification of breed-dependent SNPs associated with feed efficiency has two uses. First, beef cattle crossbreeding systems can exploit the higher feed efficiency for SNPs with dominant mode of action. Second, the use of breed-dependent SNP information may improve selection and breeding within-breeds. The identification of diet-dependent SNPs associated with feed efficiency supports breeding and selection strategies optimized for specific feeding managements. In the absence of significant interactions, significant SNP association uncover general genetic variants that are associated with feed efficiency regardless of breed or diet and that are useful across beef cattle breeding systems.

A total of 137 significant genomic associations with feed efficiency indicators, representing 93 SNPs, were detected. Associations were identified on all chromosomes with the exception of BTAs 21, 26, and 27. Ten or more associations were observed on BTAs 6, 8, 12, 15, and 17 (approximately 42% of the significant associations). Although the later chromosome had the highest number of associations (15), only three SNPs were located in gene regions.

There was a significant breed-dependent association between RFI, EI and RIG, with rs29024448 (BTA 17) and breed that indicates that this SNP has a breed-dependent mode of action (Table 7). This SNP is located within 2 kb of the replication factor C (activator 1) 5, 36.5 kDa gene (*RFC5*). The additive estimate of the minor allele G was consistent across the three indicators, with purebred SM being more efficient than 3/4AN steers. This SNP is located within a QTL region for RFI previously reported [46]. There was a significant breed-dependent association of rs110280556 with RADG and RIG (Table 7). Purebred SM steers carrying the *UNC5C* G > A allele on BTA 6 had higher feed efficiency than 3/4AN and AN. On BTA 12, rs109291606 and rs41611457, that map to ecto-NOX disulfide-thiol exchanger 1 (*ENOX1*), had a significant association with EG in a general and diet-dependent, respectively (Tables 6 and 8, respectively). All diets with the exception of Diet E (lower total net energy) had higher feed efficiency in steers carrying the variant G. This gene acts on intracellular redox homeostasis, exhibiting cyclic NADH oxidase activity [47]. Feed efficiency maybe associated with rs41611457 through disruption of the normal NADH oxidase activity because this enzyme is important for the production of reactive oxygen species (ROS). Reduction of ROS is related with limitations on energy expenditure in rats [48]. On BTA 12, two SNPs that map to the dachshund homolog 1 gene (*DACH1*) showed breed-dependent associations: rs41625438 with RIG, and rs42456314 with RFI, EI, and RIG. On BTA 15, rs41620774, rs108964818, and rs109709275 corresponding to ELMO/CED-12 domain containing 1 (*ELMOD1*), Lys-Asp-Glu-Leu containing 1-like gene (*KDELC2*), and GRAM domain containing 1B (*GRAMD1B*), respectively, had significant general associations with feed efficiency (Table 6). For *KDELC2*, the allele C > T had dominant mode of action and increased feed efficiency. Consistent across RADG, EG, and RIG, the minor allele C in *ELMOD1* had higher RADG and EG, whereas the minor allele G in *GRAMB1B* had higher RFI.

From the single SNP analyses, 29 genes located on 18 BTAs were associated with feed efficiency indicators (Table 4). In particular, the significant SNPs for ciliary neurotrophic factor receptor (*CNTFR*), CUB and Sushi multiple domains 2 (*CSMD2*), ELMO/CED-12 domain containing 1 (*ELMOD1*), glypican 5 (*GPC5*), Lys-Asp-Glu-Leu containing 1-like (*KDELC2*), replication factor C (activator 1) 5, 36.5 kDa (*RFC5*), transmembrane protein 40 (*TMEM40*), and unc-5 homolog C (*UNC5C*) genes were associated with two or more indicators (Table 9). Located on BTA 22, *TMEM40* had the highest number of associations (seven) across indicators. The same SNP in this gene (rs108942504) exhibited significant diet-by-SNP association with RIG (Table 8), and significant breed-by-SNP (Table 7) and diet-by-SNP (Table 8) associations with both EI and RFI, with the minor allele G showing lower efficiency. For the breed interaction, purebred SM animals had higher feed efficiency than composite SM. For the diet interaction, feedlot steers fed Diet E had higher feed efficiency than those fed any diet. All seven associations were consistent, showing that the allele G is associated with lower feed efficiency. Located on BTA 8, rs109500421 on *CNTFR* was associated with RFI and EI. This gene is known to regulate cell activity, participating in cytokine-cytokine receptor interaction, and in the Janus kinase/signal transducers and activators of transcription (JAK/STAT) pathway [42]. The relationship between *TMEM40* and *CNTFR* and feed efficiency may be related to the role of these genes in the transport of substances that regulates energy expenditure inside the cell.

**Table 9:** SNPs that have associations with multiple feed efficiency indicators

**SNP**	**BTA**	**Gene**	**Indicator (type of association**^**1**^**)**
rs42320097	2	-	RFI (d), EI (d) and RIG (d)
rs110742206	3	*CSMD2*	RADG (b) and EG (b)
rs41654149	4	-	RFI (g), EI (g) and RIG (g)
rs109158476	5	-	RFI (b) and EI (b)
rs109452133	6	-	RFI (d) and EI (d)
rs110280556	6	*UNC5C*	RADG (b) and RIG (b)
rs41663978	6	-	RFI (g, d) and EI (g, d)
rs43453950	6	-	RFI (d) and EI (d)
rs109500421	8	*CNTFR*	RFI (g) and EI (g)
rs110922588	8	-	RFI (g), EI (g) and RIG (g)
rs42378531	9	-	RFI (d) and EI (d)
rs109945988	11	-	RADG (g) and EG (g)
rs41256074	11	-	RFI (d) and EI (d)
rs42456314	12	*GPC5*	RFI (b), EI (b) and RIG (b)
rs110732787	13	-	RADG (g) and EG (g)
rs108964818	15	*KDELC2*	RADG (g), EG (g) and RIG (g)
rs41620774	15	*ELMOD1*	RADG (g) and EG (g)
rs41660789	15	-	RFI (d) and EI (d)
rs41634631	16	-	RFI (g) and EI (g)
rs110479395	17	-	RFI (d) and EI (d)
rs110522962	17	-	EG (g) and RIG (g)
rs111010038	17	-	RFI (g), EI (g) and RIG (g)
rs29024448	17	*RFC5*	RFI (b), EI (b) and RIG (b)
rs41854727	17	-	RFI (b), RADG (b) and RIG (b)
rs41856111	18	-	RFI (d) and RIG (d)
rs43238631	20	-	RFI (d) and EI (d)
rs108942504	22	*TMEM40*	RFI (g, b, d), EI (g, b, d) and RIG (d)
rs109863480	24	-	RFI (b) and RIG (b)
rs41619246	29	-	EI (d) and RIG (d)

^1^*g* general SNP association, *b* breed-by-SNP association, *d* diet-by-SNP association.

For rs41634631 and rs111010038, similar trends were observed for both RFI and EI (Additional file 1: Table S1). Genotypes CT and TT in rs41634631 and genotype AA in rs111010038 were associated with higher feed efficiency. Located on BTA 16, rs41634631 is within 200 kb downstream the H2.0-like homeobox (*HLX*) and molybdenum cofactor sulphurase C-terminal domain containing 1 (*MOSC1*) genes. For RADG and EG, rs109945988 (Additional file 1: Table S1) presented similar trends for both indicators such that feedlot steer with genotype TT had lower efficiency than genotypes GT and GG. This SNP is located on BTA 11, 60 kb upstream the latent tranforming growth factor beta binding protein 1 gene (*LTBP1*). For SNPs not mapped to genes interacting with diet, two polymorphisms showed high divergence between diets (Additional file 1: Table S3). For instance, the minor allele A for rs41619246 was associated with higher feed efficiency in steers fed diet C for both RIG and EI, when compared to the other diets. This SNP (BTA 11) is located with 200 kb of the genes p21 protein (Cdc42/Rac)-activated kinase 1 (*PAK1*), aquaporin 11 (*AQP11*), chloride channel, nucleotide-sensitive, 1A (*CLNS1A*), and remodeling and spacing factor 1 (*RSF1*). In contrast, for steers fed the same diet C, the minor allele C for rs41256074 is associated with lower feed efficiency by increasing both RFI and EI. This SNP is located approximately 100 kb upstream the B-cell CLL/lymphoma 11A (zinc finger protein) gene (BCL11A) on BTA 11.Steers with genotypes AA and TT, for rs41619246 and rs41256074, respectively, showed higher feed efficiency in feedlot beef production systems that use dry-rolled corn and corn gluten feed diets.

Genomic regions harboring QTL associated with RFI have been reported on all bovine chromosomes except BTAs 27 and X [10-14,46]. Significant associations with RFI were identified for 26 SNPs on 16 BTAs (Additional file 1: Tables S1, S2, and S3), with a higher concentration on BTAs 5, 6 and 17, with three, three, and four SNPs, respectively. The three SNPs on BTA 5 were associated with RFI in a breed-dependent manner. These SNPs are located between within two QTL regions for RFI that have been reported [14]. Of the three SNPs, rs110425294 (Table 7) is located approximately 675 kb upstream a region previously reported for DMI [49], and falls in the intronic region of the advillin gene (*AVIL*). The protein encoded by this gene acts as a positive regulator of neuron projection development [50]. Among feedlot steers carrying the A > G allele, 3/4SM steers had higher feed efficiency compared to ANSM. Among the SNPs on BTA 6, rs41663978 (Additional file 1: Table S3) had significant diet-dependent association. This SNP is located 8 Mb from the QTL peak location previously associated with DMI [12]. Feedlot steers that carry the C > A substitution and fed Diet E had higher RFI relative to Diet C. Of the four SNPs associated with RFI on BTA 17, rs111010038 had significant general association (Additional file 1: Table S1), rs41854727 and rs29024448 had breed-dependent associations (Additional file 1: Table S2), and rs41856111 had a diet-dependent association (Additional file 1: Table S3). Within 2 kb of the 5′UTR of the *RFC5* gene, rs29024448 is located between two regions previously associated with RFI [11,46]. For the association between rs111010038 and RFI, steers homozygous for the minor allele A have higher efficiency than steers AC and CC. In addition to these SNPs, many of the other SNPs associated with RFI in our study are located on or close (within 6 Mb) to regions previously reported, including rs109500421 on BTA 8 [46], rs41256074 on BTA 11 [11], rs109198879 on BTA 13 [12], rs41660789 on BTA 15 [13], rs41856111 on BTA 18 [46], rs43238631 on BTA 20 [46], and rs29018901 [46], and rs109863480 [13] on BTA 24.

Considering only the general association results (Additional file 1: Table S1), the minor allele had a favorable additive deviation for many SNPs (negative for RFI and EI, and positive for RADG, EG, and RIG). For instance, of the total 47 significant general associations across the indicators studied, almost half (21 associations) had the minor allele associated with higher feed efficiency. The association of these SNPs was consistent across indicators. For example, the minor allele of rs109500421 had a favorable association with RFI and EI, whereas the one for rs111010038 had a favorable association with RFI, EI and RIG. In addition, the minor alleles of rs41620774, rs110522962, and rs108964818 had favorable associations with for RADG and EG, for EG and RIG, and for RADG, EG, and RIG, respectively. This result indicates that the alleles associated with higher feed efficiency are not well represented in the population.

### Haplotype association analysis

The complete list of haplotypes associated with feed efficiency indicators at P-value < 0.001 is presented in (see Additional file 1: Table S4). The haplotypes associated with feed efficiency at P-value < 0.0001 and the contrast between the alleles with most extreme additive estimates for each haplotype across indicators are presented in Table 10.

**Table 10:** **Contrast between alleles with the minimum and maximum additive estimates of haplotypes associated**^**1 **^**with feed efficiency**

			**Allele**^**2**^			
**Indicator**	**Haplotype**	**Association**	**Minimum**	**Maximum**	**Contrast**^**3**^	**P-value**
RFI	H03	Diet-dependent	TT - Diet B	CT - Diet C	1.25 (0.25)	2.95E-5
	H11	Diet-dependent	GGGTC - Diet C	GGGTC - Diet D	2.76 (1.11)	1.11E-5
RADG	H18	Breed-dependent	GTTT - 3/4AN	ACTC - ANSM	0.48 (0.15)	6.01E-5
RIG	H03	Diet-dependent	CT - Diet C	TT - Diet B	1.68 (0.37)	2.24E-5
	H09	Breed-dependent	GAAATGA - SM	GCAATGA - 3/4AN	2.97 (1.08)	9.94E-5
	H18	Breed-dependent	ACTC - 3/4SM	ACTC - AN	1.51 (0.39)	8.14E-5
EI	H03	Diet-dependent	TT - Diet A	CT - Diet C	1.53 (0.32)	4.09E-6
	H11	Diet-dependent	GACTT - Diet C	GAGTC - Diet C	1.41 (0.52)	5.56E-6
	H16	General	GCCG	GTCT	0.45 (0.14)	5.10E-5

^1^P-value < 0.0001.

^2^Alleles with extreme additive estimates.

^3^Contrast of additive estimates between alleles with maximum and minimum estimates (standard error).

Of the 1,129 haplotypes studied, 32 significant associations with feed efficiency indicators were detected, representing 20 unique haplotypes and 81 SNPs. Of these, 36 SNPs are located in 22 different gene regions. Haplotype associations were identified on BTAs 1, 2, 5, 6, 7, 10, 11, 12, 15, 16, 17, 20, and 25, and BTAs 1, 10, 11, 12, and 15 had multiple haplotype blocks associated with feed efficiency.

**Figure 1:**
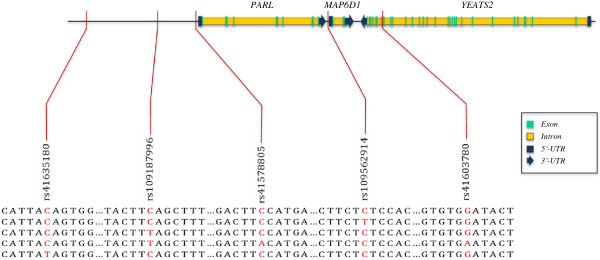
**Graphical representation of haplotype block H02 located on BTA 1.** The allelic variants of H02 are presented as the combination of the five SNPs that form this haplotype. The nitrogenous bases in red represent the possible alleles for each SNP whereas those in black are the conserved sequence flanking the SNP. The five alleles for H02 are: CCCCG, CCCTG, CTCCG, CTACA, and TCCCG. The position of the SNPs, and the number and length of exons and introns within genes were obtained in NCBI according to the Bos_taurus_UMD_3.1 assembly.

The haplotype block 2 (H02, Additional file 1: Table S4), located on BTA 1, included SNPs from three different genes, and had a significant general association with RFI. The haplotype is depicted in Figure 1 and SNPs in this block are in moderate LD (${\bar{r}}^{2}$ = 0.43). The distance between the first (rs41635180) and the last (rs41603780) SNPs is of approximately 130 kb, and rs41578805 and rs41603780, located approximately 80 kb apart, are in perfect LD (*r*^*2*^ = 1). The three genes in this block were: microtubule-associated protein 6 domain containing 1 (*MAP6D1*), presenilin associated, rhomboid-like (*PARL*), and YNL107w, ENL, AF-9, and TFIIF small subunit (YEATS) domain containing 2 (*YEATS2*). This block had two SNPs located upstream of the genes, two located within 2 kb 5′ to the genes *MAP6D1* and *PARL*, and one in an intronic region of *YEATS2* (Figure 1). These genes participate on biological processes including histone H3 acetylation (*YEATS2*), negative regulation of microtubule depolymerization (*MAP6D1*), and negative regulation of release of cytochrome c from mitochondria (*PARL*) [50]. The heme protein cytochrome c has an essential role in the mitochondrial electron transport chain, transferring electrons between Complex III and cytochrome c oxidase [51]. The efficiency of mitochondrial respiration may be affected by the availability of cytochrome c in the organelle. These results are consistent with previous reports. Low expression of the cytochrome c oxidase gene has been linked to more efficient beef cattle [52], and protein abundance is higher in efficient steers [53].

Among the haplotypes with significant association at P-value < 0.0001 (Table 10), H03 and H011 had diet-dependent associations, H09 and H18 had breed-dependent associations, and H16 had a general association with feed efficiency indicators. The latter block was significantly associated with EI, and consisted of four SNPs (BTA 15) with an average *r*^*2*^ of 0.29. Comparing the two alleles with the most extreme additive estimates, higher efficiency is expected for feedlot beef cattle carrying the haplotype allele GCCG in place of GTCT. Block H03 located on BTA 2 had an average *r*^*2*^ of 0.66 and was associated with RFI, EI, and RIG. The SNPs in this block are located in the intronic region of the F-box protein 42 gene (*FBXO42*). This gene encodes for F-box proteins, which interact with other products to act as protein-ubiquitin ligases [54]. In all three feed efficiency indicators, feedlot steers carrying the TT haplotype allele and fed diet A had higher efficiency than steers carrying the CT allele fed diet C. Block H11 was associated with RFI and EI, and is located on BTA 11 approximately 2 Mb to a QTL for RFI [11]. The highest and lowest EI were found in feedlot steers receiving diet C carrying the GACTT and GAGTC alleles, for respectively RFI and EI. Also, steers carrying the allele GGGTC and fed diet C had lower RFI compared to diet D. The SNPs in this block are in moderate LD (${\bar{r}}^{2}$ = 0.45), and the first SNP is located in the region of the sprouty-related, EVH1 domain containing 2 (*SPRED2*). This gene encodes for a protein of the Sprouty/SPRED family of proteins regulates the activation of the MAP kinase cascade [55], and *SPRED2* participates in the Jak-STAT signaling pathway, a signaling mechanism for a variety of cytokines and growth factors in mammals [42].

Four of the seven SNPs in H09 located on BTA 10 are in intronic regions of the nidogen-2 gene (*NID2*) and one in the intronic region of the DNA helicase homolog PIF1 gene (*PIF1*). This haplotype was in moderate-high LD (${\bar{r}}^{2}$ = 0.62) and had a breed-dependent association with RIG. In this block, 3/4AN steers carrying the haplotype allele GCAATGA had higher efficiency than purebred SM steers carrying GAAATGA. This haplotype is 131 kb to a QTL peak location for RFI previously reported [12]. Located on BTA 17, H18 was associated with RADG and RIG in a breed-dependent manner. Four SNPs are presented in this haplotype (Additional file 1: Table S4), with an average *r*^*2*^ of 0.61, and encompass two genes: Sin3 histone deacetylase corepressor complex component SUD3 (*SUD3)*, and serine/threonine-protein kinase TAO3 (*TAOK3*). The serine/threonine-protein kinase TAO3 participates in the MAPK signaling pathway and the JNK cascade [42,50]. For RIG, higher efficiency was observed in purebred AN steers compared to 3/4SM for the same allele ACTC. For RADG, crossbred ANSM steers carrying the favorable allele ACTC had higher feed efficiency than 3/4AN carrying the haplotype allele GTTT.

### Multi-SNP model

Feed efficiency is costly to measure on an animal basis. Genomic information can be used to develop a low-cost steer-side predictor of feed efficiency. Information on SNPs significantly associated with feed efficiency indicators was used to develop a predictor of feed efficiency in the training data set. The precision of the predictor was assessed on the validation data set.

All SNPs significantly associated at P-value < 0.001 (data not shown) were evaluated in the stepwise selection in order to consider SNPs that may have weaker associations when considered alone, and stronger associations when considered simultaneously with other SNPs. The numbers of SNPs used in the multi-SNP analyses were 227, 164, 246, 219, and 143, for RFI, RADG, RIG, EI, and EI, respectively. The selected SNPs included in the final models as general or breed/diet-dependent associations for each feed efficiency indicator is presented in Table 11. The final models for RFI, RADG, RIG, EI, and EG included: 20, 19, 13, 17, and 18 SNPs, respectively. A total of 89 SNPs (P-value < 0.0001) were fitted across all indicators. Of these, 42 SNPs were previously uncovered in the single SNP analysis and the remaining (47 SNPs) represent new associations. These new associations represent ten new genes not previously associated in the single SNP analysis. The feed efficiency indicators and additional genes are: for RFI, CD3e molecule, epsilon (CD3-TCR complex) (*CD3E*), and CCR4-NOT transcription complex, subunit 6-like (*CNOT6L*); for RADG, ATP-binding cassette, sub-family A (ABC1), member 1 (*ABCA1*), and family with sequence similarity 135, member B (*FAM135B*); for RIG, fer-1-like 5 (*FER1L5*), GTPase activating protein (SH3 domain) binding protein 2 (*G3BP2*), and spectrin, beta, non-erythrocytic 2 (*SPTBN2*); for EG, ArfGAP with GTPase domain, ankyrin repeat and PH domain 1 (*AGAP1*), cyclin M2 (*CNNM2*), glypican 5 (*GPC5*), glycerol-3-phosphate dehydrogenase 1 (soluble) (*GPD1*), and WDFY family member 4 (*WDFY4*).

**Table 11:** **SNPs [within genes] selected**^**1 **^**for the multi-SNP models and goodness-of-fit by indicator**

**Indicator**	**SNPs**			**RMSE**^**2**^		**MA**^**3**^
	**General association**	**Breed-dependent association**	**Diet-dependent association**	**T**	**V**	
RFI	rs109064731	rs108942504 [*TMEM40*]	rs108942504 [*TMEM40*]	0.4324	0.4479	3.45%
	rs109551772	rs110576675	rs109116282			
	rs110922588	rs29024448 [*RFC5*]	rs109452133			
	rs111010038	rs41568366 [*CD3E*]	rs41585447			
	rs111012032	rs41659730	rs41660789			
	rs29012628	rs42456314 [*GPC5*]	rs42169106			
	rs41588990 [*CNOT6L*]		rs42540326			
	rs41654149					
RADG	rs108964818 [*KDELC2*]	rs110280556 [*UNC5C*]	rs41627953	0.0587	0.0686	14.35%
	rs108983714	rs41574319	rs41693645			
	rs109664122	rs41583989 [*DTNA*]	rs42530614			
	rs109945988	rs41590616 [*ABCA1*]	rs43474365 [*SLC12A2*]			
	rs109957444 [*FAM135B*]	rs41600243				
	rs110732787	rs41854727				
	rs41565199					
	rs41664711					
	rs43557756					
RIG	rs108964818 [*KDELC2*]	rs41662450	rs29011654	0.5624	0.6796	17.24%
	rs109449042	rs41854727	rs41612502			
	rs110007573 [*FER1L5*]	rs42198649 [*SPTBN2*]	rs43687983			
	rs110522962					
	rs29012628					
	rs41591189 [*G3BP2*]					
	rs41654149					
	rs43557756					
EI	rs109064731	rs108942504 [*TMEM40*]	rs109198879	0.3131	0.3813	17.89%
	rs109709275 [*GRAMD1B*]	rs110206384 [*F8*]	rs109250591			
	rs110122189	rs110576675	rs42378531			
	rs110922588	rs41659730	rs43453950			
	rs111010038	rs41740922				
	rs41624569	rs42456314 [*GPC5*]				
	rs42332515					
EG	rs108964818 [*KDELC2*]	rs109880264 [*GPD1*]	rs110237102	0.0491	0.0741	33.65%
	rs109945988	rs41650269	rs41611799			
	rs110222344 [*WDFY4*]	rs42250803 [*SLC7A11*]	rs43196644			
	rs110241960	rs42973170	rs43371919 [*AGAP1*]			
	rs41574883	rs43209887				
	rs41589654 [*CNNM2*]					
	rs41625303					
	rs41664218					
	rs42457639 [*GPC5*]					

^1^P-value < 0.0001.

^2^*RMSE* root means square error for the training (T) and validation (V) data sets.

^3^*MA* model adequacy = 100*%* * [1 − (*RMSE*_*T*_/*RMSE*_*V*_)].

The SNPs in the multi-SNP model mapped to several genes with known functions that could be potentially associated with efficiency. On BTA 8, rs41590616 is located on the intronic region of the ATP-binding cassette, sub-family A (ABC1), member 1 gene (*ABCA1*) and was associated with RADG in a breed-dependent manner (Table 11). This gene encodes a transporter protein in the ABC family. This large family of proteins couple ATP hydrolysis and activate the transport of several components, such as sugars, lipids, proteins, and others. In addition, this protein plays an important role in fat digestion and absorption, transporting phospholipids and cholesterol from small intestinal epithelial cells to the intracellular space, in where these substances join apolipoprotein A1, resulting in serum high-density lipoproteins (HDL [42]). On BTA 15, rs41568366 is located in the intronic region of the CD3e molecule, epsilon (CD3-TCR complex) gene (*CD3E*); a gene that encodes for a protein that acts in immune-response related biological processes, including regulation alpha-beta T cell proliferation, interleukin-2 biosynthetic process, and interleukin-4 production [50]. This SNP was associated with RFI, and is also part of the H15 that was associated with RFI and RIG. On BTA 5, rs109880264 is located on the intronic region of the glycerol-3-phosphate dehydrogenase 1 (soluble) gene (*GPD1*). This gene has NAD binding molecular function [50] and plays a critical role in the glycerophospholipid metabolism pathway [42]. The protein encoded by this gene catalyzes the reduction of dihydroxyacetone phosphate (DHAP) to glycerol-3-phosphate (G3P), and simultaneously coverts reduced nicotine adenine dinucleotide (NADH) to nicotinamide adenine dinucleotide (NAD^+^[56]).

### SNP and haplotype validation

The SNPs and haplotypes significantly (P-value < 0.0001) associated with feed efficiency indicators on the training data set were evaluated in the validation data set. Individual SNP and haplotype associations were confirmed in the validation data set at P-value < 0.05. The less stringent P-value threshold reflects the limited number of tests that are validated, the stringent threshold used in the training data set, and the expected lower false positive rate of validation tests. For the multi-SNP models, validation was assessed by the goodness-of-fit of the selected SNPs from the training on the validation data set.

For the single-SNP analysis, the results for the validated SNPs are summarized in Additional file 1: Tables S1, S2, and S3, for SNPs with general, breed-dependent, and diet-dependent associations, respectively. A total of 17 SNPs were validated (P-value < 0.05) in the single-SNP analysis, with RFI, RADG, RIG, EI, and EG having, respectively, four, one, five, six, and one SNPs validated. These 17 associations represent 11 unique SNPs and four unique genes. The modest number of steers in the validation data set hindered the statistical power to confirm more SNPs.

The associations of rs109452133 and rs41856111 with feed efficiency were validated. On BTA 6, rs109452133 was associated with RFI and EI, and is located within 200 kb of several genes, such as the spondin 2, extracellular matrix protein (*SPON2*), C-terminal binding protein 1 (*CTBP1*), macrophage erythroblast attacher (*MAEA*), KIAA1530 ortholog (*KIAA1530*), stem-loop binding protein (*SLBP*), transmembrane protein 129 (*TMEM129*), and transforming, acidic coiled-coil containing protein 3 (*TACC3*) genes. On BTA 18, rs41856111 was associated with RFI and RIG. This SNP is located 70 kb downstream the v-maf musculoaponeurotic fibrosarcoma oncogene homolog (avian) gene (*MAF*) and approximately 1.5 Mb to a genome region previously associated with RFI [46]. Of the other five validated SNPs not mapped to genes, four were associated with RIG. Three SNPs had breed-dependent associations (rs109195623, rs41662450, and rs109137042), and rs42530614 had diet-dependent association. This SNP is located on BTA 1, 99 kb downstream from craniofacial development protein 2 (*CFDP1*), and steers carrying C alleles had higher efficiency when fed diet C compared to diet E. For the other three SNPs that had breed-dependent association with RIG, rs109195623 (BTA 3) is located 242 kb upstream the nuclear factor I/A gene (*NFIA*), rs41662450 (BTA 9) is within an uncharacterized loci 8 kb downstream the erythrocyte membrane protein band 4.1-like 2 gene (*EPB41L2*), and rs109137042 (BTA 10) is within 200 kb of the secretory carrier membrane protein 1 (*SCAMP1*), lipoma HMGIC fusion partner-like 2 (*LHFPL2*), and arylsulfatase B (*ARSB*) genes. The association between EG and rs110522962 was also validated. This SNP is located on BTA 17, approximately 100 kb upstream the predicted histone chaperone anti-silencing function-1 homolog B gene (*ASF1B*), and feedlot steers carrying the C > T substitution had higher EG and thus higher efficiency.

The SNP that maps to *TMEM40* (rs108942504) was validated in five analyses, having a general association with RFI and RADG, a breed-dependent association with RFI and EI, and a diet-dependent association with EI. The other three gene-mapped validated SNPs were rs109053103, rs108964818, and rs42072585 and are harbored in genes bridging integrator 2 (*BIN2)*, *KDELC2*, and ceroid-lipofuscinosis, neuronal 3 (*CLN3*), respectively. These SNPs had validated breed-dependent association with EI, general association with RADG, and diet-dependent association with EI, respectively. Although the SNPs in *KDELC2* and *CLN3* are located in introns, rs109053103 is within 2 kb 5′ of *BIN2*. *CLN3* is located on BTA 25 and encodes for a minor lysosomal membrane protein. Steers carrying allele G and receiving diet E had lower EI and thus higher efficiency.

The results of the haplotype analysis validation are provided in Additional file 1: Table S4. A total of seven haplotype associations were validated (P-value < 0.05), with RFI, RADG, RIG, and EI having two, one, three, and one blocks confirmed, respectively. These seven haplotype associations encompass 29 unique SNPs, seven unique genes, and six unique blocks. Two of the seven confirmed haplotypes include SNPs not mapped to genes. These are block H06, that has a general association with RADG, and H20, that has a diet-dependent association with RIG.

The validated haplotypes H07 was associated with multiple indicators. Block H07 had a breed-dependent association with RFI and EI and included four SNPs that are in moderate to low LD (${\bar{r}}^{2}$ = 0.21), in which three of them map to the intronic region of the myosin IXA gene (*MYO9A*) located on BTA 10. This promising haplotype is also located in a region previously associated with RFI [13]. Supporting the previous discussion regarding H02, this haplotype was validated and showed general association with RFI. Six SNPs located in the intronic regions of the protein phosphatase 1, regulatory subunit 12A gene (*PPP1R12A*), and one not mapped to any gene, formed H04 located on BTA 5. This gene encodes for a protein that acts in biological processes related to motility, such as vascular smooth muscle contraction, focal adhesion, and regulation of actin cytoskeleton [50]. This haplotype showed a breed-dependent association with RIG.

The validation of the multi-SNPs models was assessed by comparing the model adequacy between the training and the validation data sets using the RMSE. Low differences between the RMSE of both models, relative to the largest RMSE (corresponding to the validation data set) indicated comparable multi-SNP model adequacy across data sets. The relative differences in multi-SNP model prediction are presented in Table 11. The set of SNPs selected using the training data set fitted and predicted well in the training data set. For RFI, the loss in prediction when the training multi-SNP model was applied to the validation data set was 3.45%. This result indicates that RFI may be well predicted using the set of SNPs presented in Table 11. Likewise, there was limited loss in prediction accuracy for RADG, RIG, and EI amounting to 14.35%, 17.24%, and 17.89%, respectively. The higher loss in model fit for EG suggests that other factors not included in the model play an important role in predicting this feed efficiency indicator.

### Functional and gene networks analyses

A summary of the results from the functional enrichment analysis is presented in Table 12. For each functional annotation cluster that had an enrichment score > 3 (geometric mean of functional category P-values < 0.001), the most significant Gene Ontology molecular function, biological process and KEGG pathway categories are listed. In some clusters, only one type of category is present (e.g. general association cluster enrichment score = 11.39) meanwhile in other clusters all three types of category are present (e.g. general association, cluster enrichment score = 3.6). Genes within the enriched categories are presented in (see Additional file 1: Table S5).

**Table 12:** **Enriched**^**1 **^**functional annotation clusters and most enriched Gene Ontology and KEGG categories by SNP association**

**SNP association**	**Cluster score**	**Category**	**Name**	**Number of genes**	**P-value**
General	11.39	Molecular Function FAT	nucleotide binding	54	1.4E-19
	3.6	Biological Process FAT	protein amino acid phosphorylation	16	3.4E-05
		KEGG Pathway	MAPK signaling pathway	11	2.1E-04
		Molecular Function FAT	protein serine/threonine kinase activity	12	4.1E-04
	3.02	Molecular Function FAT	substrate specific channel activity	18	4.0E-09
		Biological Process FAT	ion transport	17	6.5E-06
Breed-dependent	16.11	Molecular Function FAT	nucleotide binding	76	3.5E-26
		Biological Process FAT	protein amino acid phosphorylation	27	7.8E-11
	9.83	Biological Process FAT	phosphorus metabolic process	35	7.2E-13
		Molecular Function FAT	protein kinase activity	25	2.3E-09
	5.42	Biological Process FAT	ion transport	32	2.3E-13
		Molecular Function FAT	ion channel activity	21	5.4E-10
	4.35	Biological Process FAT	membrane invagination	9	7.4E-06
Diet-dependent	16.71	Molecular Function FAT	nucleotide binding	65	2.1E-26
		Biological Process FAT	phosphorus metabolic process	33	5.0E-15
	3.23	Molecular Function FAT	metallopeptidase activity	12	1.5E-07
		Biological Process FAT	proteolysis	14	7.0E-03

^1^Cluster enrichment score > 3. The most significant Gene Ontology molecular function, biological process and KEGG pathway categories within each cluster are listed.

Nine clusters of Gene Ontology categories and KEGG pathways that had an enrichment score > 3 were identified among the genes encompassing general, breed-dependent, and diet-dependent SNPs. These clusters included the Gene Ontology molecular functions of nucleotide binding, protein kinase activity, and metallopeptidase activity, the Gene Ontology biological processes of ion transport, phosphorus metabolic process, membrane invagination, and proteolysis, and the MAPK signaling KEGG pathway.

The enrichment of nucleotide binding is consistent with reports that have associated nucleotide binding proteins with endocrine diseases, metabolic syndrome, and type II diabetes [57]. Similarly, protein amino acid phosphorylation has been associated with hepatic function [58]. Many of the categories encompassed in the enriched clusters (e.g. ion transport, membrane invagination) are associated with the catalysis of diffusion of substances through the cell membrane [50], a process required for the digestion, absorption of nutrients, cell growth, and survival. The three biological processes enriched among the SNPs that have breed-dependent associations encompassed general cellular events, and secretion plays an essential role in digestion and absorption of nutrients. Other promising molecular functions identified include metalloendopeptidase activity and protein kinase activity.

**Figure 2:**
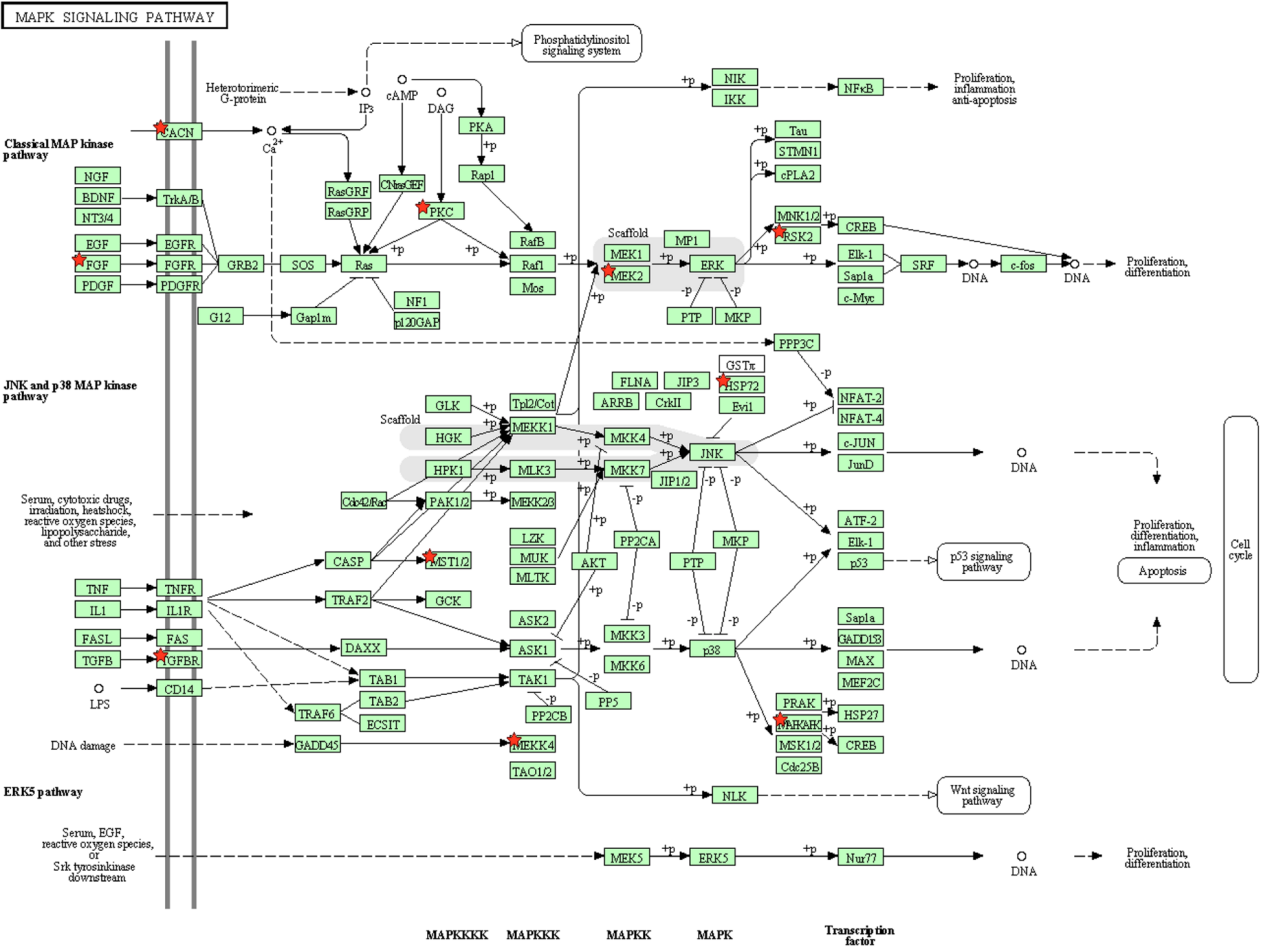
**Identification of the genes in the MAPK signaling pathway that harbor or are in the close proximity of SNPs associated with feed efficiency.** Pathway components marked with a star harbor or are in the proximity of SNPs associated with feed efficiency.

**Figure 3:**
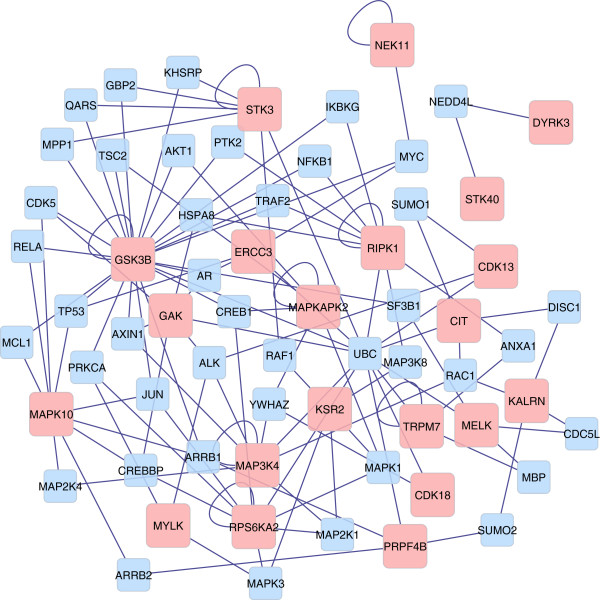
**Network of genes associated with feed efficiency that has protein serine/threonine kinase activity.** Interaction between the significant genes from the functional analysis of feed efficiency (P-value < 0.001). Genes significantly associated with feed efficiency (target genes; P-value < 0.01) are represented by pink nodes, whereas those in blue represent intermediate genes. The size of the network nodes from the target genes is a function of the P-values from the association analyses, in which larger nodes indicate more significant P-values. Target genes with self-edges indicate genes with self-regulation activity.

The identification of MAPK signaling pathway genes that either harbor or in the close proximity of SNPs are depicted in Figure 2. These genes are distributed along the pathway. The network of genes characterized by the protein serine/threonine kinase activity function is depicted in Figure 3. The pink nodes represent the target genes, whereas the blue nodes are the intermediate genes generated in the pathway analysis. In addition, the size of the nodes of the target genes is inversely proportion to their P-values. The gene with the greatest number of edges was the glycogen synthase kinase 3 beta (*GSK3B*). This gene is located on BTA 1, and encodes for a serine-threonine kinase protein involved in several metabolic and disease-related pathways [42]. Although known for acting on regulation of cell proliferation, serine/threonine kinases may also work as regulator of nutrient storage in white adipose tissue and skeletal muscle tissue [59]. Consistent with feed efficiency processes, genes encoding for proteins with serine/threonine kinase activity regulate rates of glucose uptake, fatty acid synthase transcription, and glycogen synthesis [59]. In addition to these molecular functions, the biological processes of phosphate metabolic process, phosphorus metabolic process, and protein amino acid phosphorylation were enriched for the genes of the SNPs showing diet-by-SNP association. The functional and network analyses were able to provide general roles of the genes acting on feed efficiency. Furthermore, these results show that many factors, as result of complex interactions between genes, act together in order to define feed efficiency.

### Overall performance of EI and EG

The two complementary one-step indicators to assess feed efficiency, EI and EG, were responsible for the discovery of 49 unique SNPs (Additional file 1: Tables S1, S2, and S3). Many of these SNPs were also uncovered by the traditional two-step indicators RFI and RADG.

The combined number of significant associations (P-value < 0.0001) for EI and RFI was 63, and for EG and RADG was 37. For EI and RFI, 42 SNPs were simultaneously uncovered in these two indicators, whereas the number of overlapped associations between EG and RADG was ten. The trend (direction of the estimate) and type of association (general or breed/diet dependent) of each of these associations were similar when comparing the results of the one-step indicators with their two-step counterparts (EI with RFI, and EG with RADG). In addition, the comparable genetic parameters estimated for the pairs EI/RFI and EG/RADG indicate that the one-step indicators of feed efficiency account for the same portion of phenotypic and genetic variation accounted for the two-step indicators. The benefit in using EI/EG in place of RFI/RADG may be better observed comparing the RMSE of the multi-SNP (Table 11) and null (without SNPs; data not shown) models for these indicators using the training data set. The estimated RMSE of the null models for RFI, RADG, EI, and EG, were 0.5820, 0.2492, 0.4731, and 0.2420, respectively. In all cases, the RMSE values of the one-step indicators were smaller than those for the two-step indicators. Although similar values were observed between EG and RADG, the RMSE values of the null and multi-SNP models for RFI were 23% and 38% higher than those for EI, respectively. Therefore, the high number of overlapping significant associations, the similar genetic parameter estimates, and the comparable (for EG and RADG) and favorable RMSE values (for EI over RFI) indicate that the one-step indicators (EI and EG) may at least mimic the performance of the two-step indicators (RFI and RADG) in association studies for feed efficiency in feedlot beef cattle.

## Conclusions

Genomic SNPs and haplotypes associated with feed efficiency indicators in feedlot beef steers were identified. General, breed-dependent, and diet-dependent associations were characterized and validated. These findings support both general and targeted selection decisions. Single SNPs and haplotypes showed significant association with all the five feed efficiency measures considered. Although many SNPs were associated in a general manner, other SNPs showed significant associations dependent on the breed of the animals or on the diet provided. A multi-SNP panel that can be used to predict feed efficiency was developed for each indicator including SNPs with general, and breed and diet-dependent associations. The SNP panel developed for RFI showed robust results, indicating that the set of SNPs selected can be used across breeds and diets. The complementary one-step indicators of feed efficiency (EI and EG) had comparable genetic variance than traditional two-step indicators (RFI and RADG). The unique and overrepresented SNPs, haplotypes, and genes identified for each group of indicators suggest that the one-step indicators offer complementary description of feed efficiency that can be exploited for genome-enabled selection purposes. Functional and network analysis uncovered molecular functions and biological processes enriched among the genes associated with feed efficiency. In addition, the diet- and breed-dependent genomic associations can be exploited in different production systems.

## Competing interests

The authors declare that they have no competing interests.

## Authors’ contributions

NVLS compiled the data, performed data processing, performed statistical analyses, contributed to the interpretation of results, and drafted the manuscript. JEB obtained funding for the study and helped in the drafting of the manuscript. DBF obtained funding for the study and helped in the drafting of the manuscript. DGP performed the genetic parameter analysis and helped in the drafting of the manuscript. BRS performed the gene annotation of the SNPs in the platform, and functional analyses. SRZ obtained funding for the study, participated in its conception, analysis, interpretation of results, and contributed to the final version the manuscript. All authors have read and approved the final version of this manuscript.

## Supplementary Material

Additional file 1: Table S1 Additive and dominance estimates of SNPs that have general association with feed efficiency; **Table S2.** Additive estimates of SNPs that have breed-dependent association with feed efficiency; **Table S3.** Additive estimates of SNPs that have diet-dependent association with feed efficiency; **Table S4.** Description of the haplotype blocks significantly associated with feed efficiency; **Table S5.** Enriched functional categories, Gene Ontology (GO) terms, and genes by SNP association. Click here for file
