# Supplementary material for: Single nucleotide polymorphisms and haplotypes associated with feed efficiency in beef cattle
Source: BMC Genet. 2013 Sep 25;14:94. doi: 10.1186/1471-2156-14-94 (PMC3819741; doi:10.1186/1471-2156-14-94)
Supplement: Additional file 1: Table S1 — Additive and dominance estimates of SNPs that have general association with feed efficiency; Table S2. Additive estimates of SNPs that have breed-dependent association with feed efficiency; Table S3. Additive estimates of SNPs that have diet-dependent association with feed efficiency; Table S4. Description of the haplotype blocks significantly associated with feed efficiency; Table S5. Enriched functional categories, Gene Ontology (GO) terms, and genes by SNP association. [file 1471-2156-14-94-S1.docx]

**Suppl. Table 1**. Additive and dominance effects^1^ of markers directly associated with feed efficiency

| Trait | Marker | BTA | SNP | Gene Symbol | Gene Name | Additive Effect | Dominance Effect | P-Value | Final |
| --- | --- | --- | --- | --- | --- | --- | --- | --- | --- |
| RFI | rs41654149 | 4 | C/T* |  |  | 0.03±0.04 | -0.23±0.05 | 1.09E-05 |  |
|  | rs41663978 | 6 | A*/C |  |  | 0.18±0.04 | 0.03±0.05 | 7.14E-05 |  |
|  | rs109500421 | 8 | C/T* | CNTFR | ciliary neurotrophic factor receptor | -0.01±0.05 | 0.24±0.06 | 4.54E-05 |  |
|  | rs110922588 | 8 | G/T* |  |  | 0.01±0.04 | -0.24±0.05 | 8.77E-06 |  |
|  | rs41634631 | 16 | C*/T |  |  | 1.03±0.23 | -1.09±0.23 | 1.57E-05 |  |
|  | rs111010038 | 17 | A*/C |  |  | -0.74±0.20 | 1.03±0.21 | 8.16E-07 |  |
|  | rs108942504 | 22 | A/G* | TMEM40 | transmembrane protein 40 | 0.36±0.10 | -0.09±0.11 | 9.04E-06 | yes |
| RADG | rs42433916 | 7 | G/T* |  |  | 0.02±0.01 | -0.02±0.01 | 7.40E-05 |  |
|  | rs109945988 | 11 | G/T* |  |  | -0.10±0.03 | 0.12±0.03 | 2.71E-05 |  |
|  | rs41664711 | 11 | A*/G |  |  | 0.01±0.01 | -0.04±0.01 | 2.95E-05 |  |
|  | rs110732787 | 13 | A*/G |  |  | -0.04±0.02 | -0.08±0.02 | 3.69E-05 |  |
|  | rs41565199 | 14 | C*/T |  |  | -0.02±0.01 | 0.02±0.01 | 2.63E-05 |  |
|  | rs41627953 | 14 | C*/T |  |  | -0.04±0.01 | -0.01±0.01 | 8.20E-05 |  |
|  | rs108964818 | 15 | C/T* | KDELC2 | KDEL (Lys-Asp-Glu-Leu) containing 1-like | 0.35±0.06 | 0.34±0.06 | 3.44E-08 | yes |
|  | rs41620774 | 15 | A/C* | ELMOD1 | ELMO/CED-12 domain containing 1 | 0.12±0.03 | 0.13±0.03 | 5.58E-05 |  |
|  | rs42342964 | 23 | G*/T | PAK1IP1 | PAK1 interacting protein 1 | 0.01±0.01 | 0.05±0.01 | 9.16E-06 |  |
| RIG | rs41963899 | 1 | A*/G |  |  | -0.14±0.10 | -0.45±0.11 | 2.02E-05 |  |
|  | rs41654149 | 4 | C/T* |  |  | 0.01±0.06 | 0.32±0.08 | 9.80E-05 |  |
|  | rs110922588 | 8 | G/T* |  |  | -0.01±0.06 | 0.34±0.08 | 1.86E-05 |  |
|  | rs41722387 | 14 | G*/T |  |  | -0.15±0.06 | -0.27±0.07 | 3.83E-05 |  |
|  | rs108964818 | 15 | C/T* | KDELC2 | KDEL (Lys-Asp-Glu-Leu) containing 1-like | 2.96±0.56 | 2.83±0.57 | 7.43E-07 |  |
|  | rs110522962 | 17 | C/T* |  |  | 1.10±0.26 | -0.82±0.27 | 8.18E-06 |  |
|  | rs111010038 | 17 | A*/C |  |  | 1.21±0.28 | -1.54±0.30 | 1.03E-06 |  |
|  | rs109449042 | 25 | C/T* |  |  | 1.36±0.32 | -1.25±0.33 | 9.75E-05 |  |
| EI | rs41654149 | 4 | C/T* |  |  | 0.02±0.04 | -0.22±0.05 | 2.34E-05 |  |
|  | rs41663978 | 6 | A*/C |  |  | 0.17±0.04 | 0.02±0.05 | 7.31E-05 |  |
|  | rs109500421 | 8 | C/T* | CNTFR | ciliary neurotrophic factor receptor | -0.02±0.05 | 0.23±0.06 | 5.03E-05 |  |
|  | rs110922588 | 8 | G/T* |  |  | 0.02±0.04 | -0.23±0.05 | 1.19E-05 |  |
|  | rs109709275 | 15 | A/G* | GRAMD1B | GRAM domain containing 1B | 0.05±0.05 | -0.18±0.05 | 6.29E-05 |  |
|  | rs42128656 | 15 | A*/G |  |  | -0.13±0.04 | -0.12±0.06 | 3.08E-05 |  |
|  | rs41634631 | 16 | C*/T |  |  | 0.99±0.22 | -1.05±0.22 | 1.77E-05 |  |
|  | rs111010038 | 17 | A*/C |  |  | -0.71±0.19 | 1.02±0.20 | 2.87E-07 |  |
|  | rs108942504 | 22 | A/G* | TMEM40 | transmembrane protein 40 | 0.32±0.10 | -0.05±0.11 | 2.29E-05 | yes |
|  | rs109064731 | 24 | A/G* |  |  | 0.15±0.05 | 0.25±0.06 | 7.23E-05 |  |
| EG | rs110340232 | 1 | G*/T | RAB6B | RAB6B, member RAS oncogene family | 0.01±0.01 | 0.04±0.01 | 5.07E-05 |  |
|  | rs110787048 | 4 | A*/G | DPP6 | dipeptidyl-peptidase 6 | -0.03±0.01 | -0.04±0.01 | 9.32E-05 |  |
|  | rs41574883 | 4 | A*/G |  |  | 0.07±0.01 | 0.06±0.02 | 5.47E-06 |  |
|  | rs43191790 | 4 | G/T* |  |  | 0.06±0.01 | -0.05±0.02 | 1.44E-05 |  |
|  | rs110051312 | 8 | A*/C | PTPN3 | protein tyrosine phosphatase, non-receptor type 3 | 0.04±0.03 | 0.09±0.03 | 5.46E-05 |  |
|  | rs110196238 | 8 | C*/T | PTPN3 | protein tyrosine phosphatase, non-receptor type 3 | 0.04±0.03 | 0.09±0.03 | 5.09E-05 |  |
|  | rs109945988 | 11 | G/T* |  |  | -0.13±0.03 | 0.14±0.03 | 2.59E-05 |  |
|  | rs41611457 | 12 | A/G* | ENOX1 | ecto-NOX disulfide-thiol exchanger 1 | -0.04±0.01 | 0.03±0.01 | 6.70E-05 |  |
|  | rs110732787 | 13 | A*/G |  |  | -0.03±0.02 | -0.07±0.02 | 4.29E-05 |  |
|  | rs108964818 | 15 | C/T* | KDELC2 | KDEL (Lys-Asp-Glu-Leu) containing 1-like | 0.37±0.07 | 0.37±0.07 | 4.42E-07 |  |
|  | rs41620774 | 15 | A/C* | ELMOD1 | ELMO/CED-12 domain containing 1 | 0.12±0.03 | 0.15±0.03 | 2.94E-05 |  |
|  | rs110522962 | 17 | C/T* |  |  | 0.13±0.03 | -0.10±0.03 | 2.24E-05 | yes |
|  | rs109889052 | 19 | C/T* | PIK3R6 | phosphoinositide-3-kinase, regulatory subunit 6 | -0.27±0.07 | 0.29±0.07 | 7.79E-05 |  |

^1^ Using the minor allele as reference;

^*^ Minor allele;

**Suppl. Table 2**. Additive effect^1^ of markers associated with feed efficiency in a breed-dependent manner

| Trait | Marker | BTA | SNP | Gene Symbol | Gene Name | Breed | | | | P-Value | Final |
| --- | --- | --- | --- | --- | --- | --- | --- | --- | --- | --- | --- |
|  |  |  |  |  |  | AN | 3/4 AN | AN/SM | 3/4 SM |  |  |
| RFI | rs109158476 | 5 | C*/T |  |  | -0.06±0.07 | 0.04±0.07 | 0.1±0.06 | -0.4±0.09 | 7.08E-06 |  |
|  | rs110425294 | 5 | A/G* | AVIL | advillin | -0.09±0.06 | 0.09±0.06 | 0.13±0.05 | -0.23±0.08 | 5.71E-05 |  |
|  | rs29026607 | 5 | C/T* |  |  | -0.12±0.07 | -0.02±0.07 | 0.04±0.06 | -0.51±0.1 | 8.10E-05 |  |
|  | rs110708529 | 12 | G/T* |  |  | -0.23±0.07 | -0.4±0.07 | -0.21±0.06 | -0.47±0.09 | 8.84E-05 |  |
|  | rs42456314 | 12 | A/G* | GPC5 | glypican 5 | -0.08±0.07 | -0.27±0.07 | -0.2±0.06 | -0.57±0.08 | 2.69E-05 |  |
|  | rs29024448 | 17 | G/T* | RFC5 | replication factor C (activator 1) 5, 36.5kDa | 0.14±0.06 | 0.31±0.06 | 0.05±0.05 | 0.2±0.07 | 8.32E-06 |  |
|  | rs41854727 | 17 | C/T* |  |  | 0.01±0.08 | 0.17±0.07 | -0.04±0.06 | 0.36±0.1 | 2.73E-05 |  |
|  | rs108942504 | 22 | A/G* | TMEM40 | transmembrane protein 40 | 0.05±0.13 | 0.15±0.12 | 0.17±0.1 | 0.53±0.13 | 3.07E-06 | yes |
|  | rs109863480 | 24 | C*/T |  |  | 0.06±0.07 | -0.04±0.07 | 0.06±0.06 | -0.57±0.1 | 1.26E-05 |  |
|  | rs29018901 | 24 | A*/G |  |  | 0.18±0.07 | 0.09±0.07 | 0.13±0.06 | -0.46±0.1 | 3.75E-05 |  |
| RADG | rs109808044 | 3 | A*/G | SNED1 | sushi, nidogen and EGF-like domains 1 | -0.04±0.01 | -0.08±0.01 | -0.01±0.01 | -0.03±0.01 | 2.44E-05 |  |
|  | rs110742206 | 3 | C/T* | CSMD2 | CUB and Sushi multiple domains 2 | 0.11±0.03 | 0.11±0.03 | 0.08±0.02 | 0.23±0.04 | 1.98E-05 |  |
|  | rs109320755 | 4 | C/T* |  |  | -0.04±0.01 | -0.05±0.01 | -0.02±0.01 | -0.02±0.01 | 7.15E-05 |  |
|  | rs110690110 | 5 | C*/G | ERC1 | ELKS/RAB6-interacting/CAST family member 1 | -0.03±0.01 | -0.05±0.01 | -0.02±0.01 | -0.07±0.01 | 2.49E-05 |  |
|  | rs110280556 | 6 | A*/G | UNC5C | unc-5 homolog C (C. elegans) | -0.02±0.01 | -0.03±0.01 | 0±0.01 | 0.05±0.01 | 6.65E-06 |  |
|  | rs110244477 | 8 | A*/T |  |  | 0.08±0.02 | 0.12±0.02 | 0.05±0.01 | 0.09±0.02 | 9.09E-05 |  |
|  | rs41854727 | 17 | C/T* |  |  | -0.02±0.01 | -0.03±0.01 | 0.01±0.01 | -0.04±0.02 | 2.96E-05 |  |
|  | rs41583989 | 24 | C*/T | DTNA | dystrobrevin, alpha | -0.07±0.02 | 0.04±0.02 | -0.02±0.01 | -0.04±0.02 | 9.20E-05 |  |
|  | rs41600243 | 28 | A/G* |  |  | 0.05±0.01 | 0±0.01 | 0.02±0.01 | 0±0.01 | 6.52E-05 |  |
| RIG | rs110131536 | 2 | A*/G | IGFBP5 | insulin-like growth factor binding protein 5 | 0.33±0.14 | 0.4±0.13 | 0.47±0.12 | -0.69±0.2 | 2.30E-05 |  |
|  | rs109195623 | 3 | A*/C |  |  | -0.15±0.12 | -0.28±0.12 | -0.37±0.11 | 0.82±0.21 | 7.00E-05 | yes |
|  | rs110280556 | 6 | A*/G | UNC5C | unc-5 homolog C (C. elegans) | -0.13±0.08 | -0.11±0.08 | 0.06±0.06 | 0.62±0.11 | 3.88E-05 |  |
|  | rs41613098 | 6 | C/T* |  |  | 0.14±0.12 | 0.08±0.13 | 0.29±0.1 | -1.02±0.2 | 2.66E-06 |  |
|  | rs109533642 | 7 | C*/T |  |  | 0.45±0.14 | -0.07±0.12 | -0.07±0.1 | -0.81±0.17 | 5.63E-05 |  |
|  | rs41662450 | 9 | C/T* |  |  | 0.18±0.09 | 0.01±0.09 | 0.03±0.07 | -0.59±0.11 | 3.97E-05 | yes |
|  | rs109137042 | 10 | A*/G |  |  | 0.11±0.17 | 0.14±0.18 | 0.16±0.14 | -1.61±0.31 | 5.37E-05 | yes |
|  | rs110745951 | 10 | A/G* |  |  | 0.09±0.1 | 0.07±0.1 | -0.08±0.08 | 0.28±0.11 | 8.71E-05 |  |
|  | rs109885711 | 12 | C/T* |  |  | 0.2±0.09 | -0.01±0.09 | 0.21±0.07 | 0.79±0.12 | 1.35E-05 |  |
|  | rs41624425 | 12 | C/T* |  |  | -0.2±0.15 | -0.23±0.15 | -0.19±0.13 | -1.4±0.23 | 4.75E-05 |  |
|  | rs41625438 | 12 | C/T* | DACH1 | dachshund homolog 1 (Drosophila) | 0.06±0.15 | 0.03±0.15 | 0.05±0.13 | 1.08±0.21 | 8.82E-05 |  |
|  | rs41626923 | 12 | C*/T |  |  | 0.09±0.09 | 0.03±0.09 | 0.1±0.07 | 0.81±0.12 | 4.05E-05 |  |
|  | rs42456314 | 12 | A/G* | GPC5 | glypican 5 | 0.22±0.1 | 0.41±0.1 | 0.36±0.08 | 0.9±0.12 | 2.35E-06 |  |
|  | rs43693414 | 12 | C/T* |  |  | 0.1±0.13 | 0.2±0.13 | 0.18±0.12 | 0.99±0.18 | 4.66E-05 |  |
|  | rs42206139 | 15 | A*/G |  |  | -0.31±0.11 | -0.54±0.1 | -0.21±0.08 | 0.14±0.12 | 6.66E-05 |  |
|  | rs41623603 | 16 | A*/C | CNST | consortin, connexin sorting protein | 0.08±0.1 | 0.1±0.11 | 0.19±0.09 | -0.36±0.13 | 5.13E-05 |  |
|  | rs29024448 | 17 | G*/T | RFC5 | replication factor C (activator 1) 5, 36.5kDa | -0.15±0.09 | -0.48±0.09 | -0.09±0.07 | -0.32±0.11 | 1.71E-05 |  |
|  | rs41854727 | 17 | C/T* |  |  | -0.13±0.11 | -0.34±0.11 | 0.06±0.09 | -0.56±0.14 | 2.14E-07 |  |
|  | rs109863480 | 24 | C*/T |  |  | -0.07±0.1 | -0.05±0.1 | -0.1±0.08 | 0.84±0.14 | 6.30E-06 |  |
|  | rs42666807 | 24 | C*/T |  |  | 0.17±0.1 | -0.18±0.09 | -0.04±0.08 | 0.66±0.13 | 4.41E-05 |  |
| EI | rs41659730 | 4 | A*/G |  |  | -0.27±0.07 | 0.27±0.07 | 0.1±0.05 | -0.07±0.07 | 2.87E-05 |  |
|  | rs109053103 | 5 | A*/G | BIN2 | bridging integrator 2-like | 0.28±0.08 | 0.28±0.08 | -0.01±0.06 | 0.36±0.1 | 8.57E-05 | yes |
|  | rs109158476 | 5 | C*/T |  |  | -0.06±0.07 | 0.06±0.07 | 0.11±0.06 | -0.35±0.09 | 1.52E-05 |  |
|  | rs42456314 | 12 | A/G* | GPC5 | glypican 5 | -0.1±0.07 | -0.26±0.07 | -0.21±0.06 | -0.55±0.08 | 3.13E-05 |  |
|  | rs29024448 | 17 | G*/T | RFC5 | replication factor C (activator 1) 5, 36.5kDa | 0.11±0.06 | 0.3±0.06 | 0.07±0.05 | 0.19±0.07 | 3.23E-05 |  |
|  | rs41567063 | 17 | C/T* |  |  | 0.49±0.08 | 0.22±0.07 | 0.21±0.05 | 0.12±0.08 | 2.91E-05 |  |
|  | rs108942504 | 22 | A/G* | TMEM40 | transmembrane protein 40 | 0.05±0.12 | 0.09±0.11 | 0.16±0.1 | 0.48±0.13 | 8.72E-06 | yes |
|  | rs110206384 | X | A/G* | F8 | coagulation factor VIII, procoagulant component | 0.17±0.05 | -0.07±0.05 | -0.02±0.04 | 0.01±0.07 | 5.48E-05 |  |
| EG | rs110742206 | 3 | C/T* | CSMD2 | CUB and Sushi multiple domains 2 | 0.54±0.13 | 0.46±0.12 | 0.35±0.11 | 1.08±0.17 | 7.31E-05 |  |
|  | rs41613367 | 8 | A*/G |  |  | -0.09±0.04 | -0.08±0.04 | 0.05±0.04 | -0.13±0.05 | 9.24E-05 |  |
|  | rs41620815 | 8 | A*/C |  |  | -0.31±0.06 | -0.25±0.05 | -0.19±0.05 | -0.45±0.07 | 3.49E-05 |  |
|  | rs42250803 | 17 | A*/G | SLC7A11 | solute carrier family 7 (anionic amino acid transporter light chain, xc- system), member 11 | 0.06±0.06 | -0.06±0.05 | 0.02±0.04 | -0.31±0.06 | 2.23E-05 |  |

^1^ Using the minor allele as reference;

^*^ Minor allele;

**Suppl. Table 3**. Additive effect^1^ of markers associated with feed efficiency in a diet-dependent manner

| Trait | Marker | BTA | SNP | Gene Symbol | Gene Name | Diet | | | | P-Value | Final |
| --- | --- | --- | --- | --- | --- | --- | --- | --- | --- | --- | --- |
|  |  |  |  |  |  | A | B | C | D |  |  |
| RFI | rs42320097 | 2 | A*/G |  |  | -0.06±0.07 | -0.2±0.07 | -0.61±0.1 | -0.04±0.07 | 8.48E-05 |  |
|  | rs109452133 | 6 | A/G* |  |  | -0.12±0.07 | -0.02±0.06 | 0.26±0.07 | 0.07±0.06 | 1.07E-06 | yes |
|  | rs41663978 | 6 | A*/C |  |  | 0.07±0.05 | 0.1±0.05 | 0.19±0.06 | 0.08±0.05 | 9.54E-05 |  |
|  | rs43453950 | 6 | A*/G |  |  | -0.05±0.05 | 0±0.05 | 0.31±0.07 | 0.01±0.06 | 3.25E-05 |  |
|  | rs42378531 | 9 | C/T* |  |  | -0.07±0.05 | 0.13±0.05 | 0.14±0.06 | 0.09±0.05 | 5.31E-05 |  |
|  | rs41256074 | 11 | C*/T |  |  | -0.09±0.1 | 0.07±0.09 | 0.5±0.13 | -0.22±0.1 | 5.24E-05 |  |
|  | rs109198879 | 13 | A/G* |  |  | 0.05±0.05 | 0.2±0.05 | 0.13±0.06 | 0.03±0.05 | 8.88E-05 |  |
|  | rs41660789 | 15 | A*/G |  |  | -0.14±0.06 | -0.09±0.06 | 0.12±0.06 | -0.21±0.06 | 2.02E-05 |  |
|  | rs110479395 | 17 | C*/T |  |  | -0.01±0.08 | 0.07±0.08 | 0.53±0.11 | -0.07±0.08 | 2.88E-05 |  |
|  | rs41856111 | 18 | C/T* |  |  | -0.06±0.07 | -0.02±0.07 | -0.24±0.07 | 0.13±0.07 | 2.94E-05 | yes |
|  | rs43238631 | 20 | A/G* |  |  | 0.05±0.06 | 0.01±0.05 | -0.35±0.08 | 0.11±0.05 | 7.60E-05 |  |
|  | rs108942504 | 22 | A/G* | TMEM40 | transmembrane protein 40 | 0.27±0.08 | 0.28±0.08 | 0.29±0.09 | 0.22±0.08 | 1.66E-05 |  |
| RADG | rs43474365 | 7 | A/G* | SLC12A2 | solute carrier family 12 (sodium/potassium/chloride transporters), member 2 | 0±0.01 | -0.03±0.01 | 0.01±0.01 | -0.03±0.01 | 4.11E-05 |  |
| RIG | rs41609661 | 1 | G*/T |  |  | -0.38±0.07 | -0.45±0.07 | -0.41±0.08 | -0.21±0.07 | 6.72E-05 |  |
|  | rs42530614 | 1 | C*/T |  |  | 0.39±0.08 | 0.45±0.07 | 0.49±0.09 | 0.22±0.07 | 3.25E-05 | yes |
|  | rs42320097 | 2 | A*/G |  |  | 0.26±0.1 | 0.45±0.1 | 0.98±0.14 | 0.28±0.1 | 4.29E-05 |  |
|  | rs29011654 | 3 | A/G* |  |  | -0.4±0.07 | -0.36±0.07 | -0.47±0.08 | -0.25±0.07 | 9.64E-05 |  |
|  | rs41856111 | 18 | C/T* |  |  | 0.14±0.1 | 0.06±0.09 | 0.37±0.1 | -0.14±0.1 | 4.81E-05 | yes |
|  | rs43687983 | 20 | A*/G |  |  | 0.46±0.08 | 0.28±0.08 | 0.13±0.1 | 0.33±0.08 | 2.99E-05 |  |
|  | rs108942504 | 22 | A/G* | TMEM40 | transmembrane protein 40 | -0.51±0.12 | -0.4±0.11 | -0.46±0.14 | -0.32±0.12 | 5.80E-05 |  |
|  | rs41612502 | 28 | A/G* |  |  | -0.23±0.11 | -0.04±0.11 | -0.12±0.13 | 0.11±0.11 | 8.98E-05 |  |
|  | rs41619246 | 29 | A*/G |  |  | 0±0.13 | 0.3±0.12 | 1.84±0.41 | -0.31±0.14 | 4.26E-05 |  |
| EI | rs42320097 | 2 | A*/G |  |  | -0.05±0.07 | -0.18±0.07 | -0.63±0.1 | -0.05±0.07 | 4.17E-05 |  |
|  | rs41593945 | 4 | A*/C | CNPY1 | canopy 1 homolog | -0.04±0.07 | -0.16±0.07 | 0.22±0.07 | -0.02±0.07 | 4.80E-05 |  |
|  | rs109452133 | 6 | A/G* |  |  | -0.11±0.06 | -0.01±0.06 | 0.26±0.07 | 0.07±0.06 | 1.06E-06 | yes |
|  | rs109976880 | 6 | A*/G |  |  | 0.09±0.06 | -0.03±0.06 | -0.19±0.06 | -0.01±0.06 | 8.42E-05 |  |
|  | rs41663978 | 6 | A*/C |  |  | 0.03±0.05 | 0.1±0.05 | 0.17±0.05 | 0.06±0.05 | 7.42E-05 |  |
|  | rs43453950 | 6 | A*/G |  |  | -0.02±0.05 | 0.02±0.05 | 0.36±0.06 | 0.02±0.05 | 6.46E-07 |  |
|  | rs110707592 | 9 | C/T* |  |  | -0.22±0.05 | -0.03±0.05 | 0.01±0.06 | -0.21±0.05 | 1.95E-05 |  |
|  | rs42378531 | 9 | C/T* |  |  | -0.09±0.05 | 0.12±0.05 | 0.16±0.06 | 0.08±0.05 | 2.30E-06 |  |
|  | rs42952059 | 9 | A/G* |  |  | -0.23±0.05 | -0.12±0.05 | 0.04±0.06 | -0.24±0.05 | 3.16E-05 |  |
|  | rs41256074 | 11 | C*/T |  |  | -0.1±0.1 | 0.07±0.09 | 0.54±0.12 | -0.23±0.1 | 5.38E-06 |  |
|  | rs41660789 | 15 | A*/G |  |  | -0.11±0.06 | -0.06±0.05 | 0.17±0.06 | -0.16±0.06 | 7.14E-06 |  |
|  | rs110479395 | 17 | C*/T |  |  | -0.01±0.08 | 0.07±0.08 | 0.51±0.11 | -0.08±0.08 | 1.21E-05 |  |
|  | rs43238631 | 20 | A/G* |  |  | 0.06±0.06 | -0.01±0.05 | -0.37±0.08 | 0.1±0.05 | 1.22E-05 |  |
|  | rs108942504 | 22 | A/G* | TMEM40 | transmembrane protein 40 | 0.21±0.08 | 0.23±0.07 | 0.24±0.09 | 0.18±0.08 | 8.28E-05 | yes |
|  | rs42072585 | 25 | A/G* | CLN3 | ceroid-lipofuscinosis, neuronal 3 | 0.18±0.06 | 0.21±0.06 | 0.36±0.06 | 0.27±0.06 | 7.02E-05 | yes |
|  | rs41619246 | 29 | A*/G |  |  | -0.01±0.09 | -0.18±0.08 | -1.41±0.27 | 0.18±0.09 | 6.83E-05 |  |
| EG | rs109291606 | 12 | G/T* | ENOX1 | ecto-NOX disulfide-thiol exchanger 1 | 0.04±0.01 | 0.04±0.01 | 0.04±0.01 | 0.02±0.01 | 8.93E-05 |  |

^1^ Using the minor allele as reference;

^*^ Minor allele;

**Suppl. Table 4**. Location, composition and LD of the haplotypes associated with feed efficiency

| Haplotype | BTA | Marker | SNP | Gene | $\bar{r}^{2}$ | Traits (Effect) |
| --- | --- | --- | --- | --- | --- | --- |
| H01 | 1 | rs41623606 | C/T | - | 0.15 | EI (b), RFI (b), and RIG (b) |
|  |  | rs43211556 | A/C | - |  |  |
|  |  | rs43210901 | A/G | - |  |  |
|  |  | rs41630314 | G/T | *GRIK1* |  |  |
| H02 | 1 | rs41635180 | C/T | - | 0.43 | RFI (m)^V^ |
|  |  | rs109187996 | C/T | - |  |  |
|  |  | rs41578805 | A/C | *PARL* |  |  |
|  |  | rs109562914 | C/T | *MAP6D1* |  |  |
|  |  | rs41603780 | A/G | *YEATS2* |  |  |
| H03 | 2 | rs43324587 | C/T | *FBXO42* | 0.34 | EI (d), RFI (d), and RIG (d) |
|  |  | rs43326092 | C/T | *FBXO42* |  |  |
| H04 | 5 | rs43426639 | C/T | *PPP1R12A* | 0.43 | RIG (b)^V^ |
|  |  | rs43426641 | G/T | *PPP1R12A* |  |  |
|  |  | rs41592931 | A/G | *PPP1R12A* |  |  |
|  |  | rs41653612 | C/T | *PPP1R12A* |  |  |
|  |  | rs41653616 | C/T | *PPP1R12A* |  |  |
|  |  | rs41566962 | C/T | *PPP1R12A* |  |  |
|  |  | rs110140052 | C/T | - |  |  |
| H05 | 6 | rs110767541 | C/T | - | 0.37 | RADG (b) |
|  |  | rs29025601 | A/G | *FAM13A* |  |  |
|  |  | rs41627896 | A/G | *FAM13A* |  |  |
|  |  | rs109998457 | A/T | - |  |  |
| H06 | 7 | rs110957342 | A/C | - | 0.21 | RADG (m)^V^ |
|  |  | rs41596177 | A/G | - |  |  |
|  |  | rs41669611 | C/T | - |  |  |
|  |  | rs42315549 | C/T | - |  |  |
| H07 | 10 | rs109668946 | A/T | *MYO9A* | 0.21 | EI (b)^V^ and RFI (b)^V^ |
|  |  | rs110829769 | A/T | *MYO9A* |  |  |
|  |  | rs41626107 | C/G | *MYO9A* |  |  |
|  |  | rs41626110 | C/T | - |  |  |
| H08 | 10 | rs108977212 | C/T | *CHP* | 0.22 | RIG (b) |
|  |  | rs109827805 | C/T | *NDUFAF1* |  |  |
|  |  | rs41584997 | A/G | - |  |  |
|  |  | rs109430556 | C/T | *RTF1* |  |  |
| H09 | 10 | rs43626304 | A/G | *NID2* | 0.60 | RADG (b) and RIG (b)^V^ |
|  |  | rs43625528 | A/C | *NID2* |  |  |
|  |  | rs43625453 | A/C | *NID2* |  |  |
|  |  | rs41588508 | A/G | *NID2* |  |  |
|  |  | rs41588507 | C/T | - |  |  |
|  |  | rs43627130 | G/T | - |  |  |
|  |  | rs43710946 | A/G | *PIF1* |  |  |
| H10 | 11 | rs29003479 | C/T | *RAB1A* | 0.22 | RIG (d) |
|  |  | rs41668653 | C/T | - |  |  |
|  |  | rs29018917 | C/T | - |  |  |
|  |  | rs41599919 | C/T | - |  |  |
|  |  | rs29015166 | A/G | *ACTR2* |  |  |
| H11 | 11 | rs110935406 | A/G | *SPRED2* | 0.45 | EI (d), RFI (d), and RIG (d) |
|  |  | rs109658327 | A/G | - |  |  |
|  |  | rs110624650 | C/G | - |  |  |
|  |  | rs110351243 | C/T | - |  |  |
|  |  | rs110249674 | C/T | - |  |  |
| H12 | 12 | rs41571241 | C/T | - | 0.33 | EG (d) and RADG (d) |
|  |  | rs43688242 | C/T | - |  |  |
|  |  | rs43081920 | A/C | - |  |  |
| H13 | 12 | rs110364209 | A/T | - | 0.27 | RADG (b) |
|  |  | rs109845266 | C/T | *TNFSF11* |  |  |
|  |  | rs110425177 | A/G | *TNFSF11* |  |  |
|  |  | rs110699038 | C/T | - |  |  |
|  |  | rs41630106 | G/T | - |  |  |
| H14 | 12 | rs109675697 | C/T | - | 0.41 | RIG (b) |
|  |  | rs110448596 | C/T | - |  |  |
| H15 | 15 | rs41568366 | A/G | *CD3E* |  | RFI (m) and RIG (m) |
|  |  | rs41568365 | G/T | *CD3E* |  |  |
| H16 | 15 | rs43291568 | A/G | - | 0.29 | EI (m), RFI (m) |
|  |  | rs43291603 | C/T | - |  |  |
|  |  | rs29014510 | C/T | - |  |  |
|  |  | rs110441482 | G/T | - |  |  |
| H17 | 16 | rs41806515 | A/G | - | 0.33 | EI (d) |
|  |  | rs41805926 | A/G | - |  |  |
|  |  | rs109248300 | A/G | - |  |  |
|  |  | rs41579702 | G/T | - |  |  |
| H18 | 17 | rs41842607 | A/G | - | 0.61 | RADG (b) and RIG (b) |
|  |  | rs41842594 | C/T | *SUDS3* |  |  |
|  |  | rs41842624 | C/T | - |  |  |
|  |  | rs41843338 | C/T | *TAOK3* |  |  |
| H19 | 20 | rs42680543 | A/G | - | 0.29 | EI (m) and RFI (m) |
|  |  | rs41946124 | C/T | *RXFP3* |  |  |
|  |  | rs41601589 | A/G | *RXFP3* |  |  |
|  |  | rs110201922 | G/T | *ADAMTS12* |  |  |
| H20 | 25 | rs110665834 | A/G | - | 0.37 | RIG (d)^V^ |
|  |  | rs41664150 | C/T | - |  |  |

^V^ Effect in which the haplotype was validated;

**Suppl. Table 5**. Functional categories and term, and enriched genes by SNP effect

| SNP effect | Cluster score | Category | Name | Number of Genes | P-value | Genes |
| --- | --- | --- | --- | --- | --- | --- |
| General | 11.39 | Molecular Function FAT | nucleotide binding | 54 | 1.4E-19 | CLPB, PDPK1, ACSS1, MAP3K4, RAVER2, RAB6B, AGAP1, SAR1B, DHX32, PRKCA, GTPBP4, PFKL, GTF2H4, G3BP2, MYH9, STK3, XPA, DNAJC27, NAV2, RIPK1, EEFSEC, SMARCA2, KALRN, ENOX1, RALYL, BLM, PFKFB3, MAPKAPK3, DNAH2, ABCA6, TDRD10, HSPA1L, IGF1R, DDX47, ABCD3, RAB11A, SNRNP35, EWSR1, DHX57, GUCY2F, MAP2K2, ALPK2, MAK, TGFBR1, MSH5, WRN, DACH1, ACACB, ABCB5, RABL2B, EPHA6, PDE2A, RPS6KA1, RAD54B |
|  | 3.6 | Biological Process FAT | protein amino acid phosphorylation | 16 | 3.4E-05 | PRKCA, GUCY2F, MAP2K2, ALPK2, MAK, TGFBR1, MAPKAPK3, STK3, IGF1R, PDPK1, MAP3K4, EPHA6, RPS6KA1, RIPK1, FGF2, KALRN |
|  |  | KEGG Pathway | MAPK signaling pathway | 11 | 2.1E-04 | PRKCA, HSPA1L, MAP3K4, RPS6KA1, MAP2K2, TGFBR1, MAPKAPK3, CACNB2, CACNB4, FGF2, STK3 |
|  |  | Molecular Function FAT | protein serine/threonine kinase activity | 12 | 4.1E-04 | PRKCA, PDPK1, MAP3K4, RPS6KA1, ALPK2, MAP2K2, MAK, RIPK1, TGFBR1, MAPKAPK3, STK3, KALRN |
|  | 3.02 | Molecular Function FAT | substrate specific channel activity | 18 | 4.0E-09 | TRPC2, GABRA3, CACNB2, CACNB4, GRIA4, KCNK2, KCNIP4, ACCN1, GABRR3, KCNK9, GRIA2, GRIN2B, RYR3, TTYH1, GRID2, KCNH8, NALCN, FGF2 |
|  |  | Biological Process FAT | ion transport | 17 | 6.5E-06 | TRPC2, GABRA3, CACNB2, CACNB4, GRIA4, KCNK2, KCNIP4, ACCN1, GABRR3, KCNK9, GRIA2, GRIN2B, RYR3, TTYH1, GRID2, KCNH8, NALCN |
| Breed-dependent | 16.11 | Molecular Function FAT | nucleotide binding | 76 | 3.5E-26 | NCBP2, ADCY1, KIF27, CLPB, FOX1, INO80, CCT2, ATP10D, SART3, PRKG1, ACSS3, TPK1, TOR3A, NUBP2, RAVER2, RASL10A, RBMS3, AGAP1, EGFR, GTPBP4, CSNK1G1, TRPM7, KIF16B, PRKCE, CLPX, STK3, PRKCB, COQ6, RFC5, PRKD1, KIF1A, NNT, SCYL1, RFC2, RIPK1, TESK2, PCCB, PCCA, MELK, ENOX1, RALYL, TDRD9, MAPKAPK3, ABCA1, ABCA6, SFRS15, SPEG, SYN3, MSI2, RAB11A, STK38L, EWSR1, GUCY2F, GPD1, FLT1, GNAO1, TGFBR1, MAPK10, DACH1, ACACB, HRNBP3, ATP13A5, RIMKLA, ICK, ABCG5, RPS6KA2, FYN, NTRK2, MYH11, PHGDH, RBM19, GRK4, GRK5, DNM2, MYH10, RBM17 |
|  |  | Biological Process FAT | protein amino acid phosphorylation | 27 | 7.8E-11 | MAPKAPK3, PRKG1, SPEG, CHRNA7, THBS1, STK38L, EGFR, GUCY2F, FLT1, CSNK1G1, TRPM7, TGFBR1, MAPK10, PRKCE, STK3, PRKCB, PRKD1, ICK, SCYL1, RPS6KA2, FYN, RIPK1, NTRK2, TESK2, GRK4, GRK5, MELK |
|  | 9.83 | Biological Process FAT | phosphorus metabolic process | 35 | 7.2E-13 | MAPKAPK3, ATP6V1B1, PRKG1, MTMR2, THTPA, SPEG, CHRNA7, THBS1, STK38L, EGFR, GPD1, PTPRK, GUCY2F, FLT1, CSNK1G1, PTPRG, TRPM7, TGFBR1, PTPRT, MAPK10, PRKCE, STK3, PRKCB, PTPN11, PRKD1, ICK, SCYL1, RPS6KA2, FYN, RIPK1, NTRK2, TESK2, GRK4, GRK5, MELK |
|  |  | Molecular Function FAT | protein kinase activity | 25 | 2.3E-09 | MAPKAPK3, PRKG1, SPEG, STK38L, EGFR, GUCY2F, FLT1, CSNK1G1, TRPM7, TGFBR1, MAPK10, PRKCE, STK3, PRKCB, PRKD1, ICK, SCYL1, FYN, RPS6KA2, RIPK1, NTRK2, TESK2, GRK4, GRK5, MELK |
|  | 5.42 | Biological Process FAT | ion transport | 32 | 2.3E-13 | PLCZ1, KCNC2, GRIK1, SLC39A11, GRIK4, SLC26A10, ATP6V1B1, CNGB3, KCNIP4, TPCN1, ATP5S, CHRNA7, SLCO3A1, SCN7A, NFATC1, KCNMA1, GABRA2, GABRG3, SLC12A2, TRPM7, ATP13A5, PRKCB, KCTD8, ACCN2, ACCN1, GABRR3, NNT, GRIA2, KCNN2, CACNA1H, CACNA1E, KCNH8 |
|  |  | Molecular Function FAT | ion channel activity | 21 | 5.4E-10 | KCNMA1, KCNC2, GABRA2, GABRG3, GRIK1, TRPM7, GRIK4, CNGB3, TPCN1, KCNIP4, KCTD8, ACCN2, ACCN1, GABRR3, GRIA2, KCNN2, CACNA1H, CHRNA7, KCNH8, CACNA1E, SCN7A |
|  | 4.35 | Biological Process FAT | membrane invagination | 9 | 7.4E-06 | AP2A2, FCGR1A, WASF2, CHRNA7, ABCA1, THBS1, ELMO1, DNM2, ELMOD1 |
| Diet-dependent | 16.71 | Molecular Function FAT | nucleotide binding | 65 | 2.1E-26 | NCBP2, CDK18, PRPF4B, DICER1, RAB1A, PIP5KL1, MAP3K4, AAK1, RBMS3, AGAP1, EIF2B2, DOCK11, CDK13, TRPM7, KIF5C, PRKCI, KIF16B, NEK11, STK3, GAK, KSR2, NAV2, RIPK1, SMARCA2, MELK, ENOX1, KALRN, DNAH11, SMARCAD1, RAB7B, PFKFB4, HDGF, POLA1, MAPKAPK2, ULK4, ABCA4, TDRD10, MUSK, STK40, DYRK3, SNRNP70, EHD1, PAPSS1, ERCC3, EWSR1, NOX4, KIF3B, FLT1, NOX5, MYO1B, AK5, DRG1, WRN, MAPK10, DACH1, UBE2L3, HRNBP3, ELAVL4, AK7, ABCG5, RPS6KA2, GSK3B, DDX50, CIT, MYLK |
|  |  | Biological Process FAT | phosphorus metabolic process | 33 | 5.0E-15 | PRPF4B, CDK18, ULK4, MAPKAPK2, MUSK, MAP3K4, STK40, AAK1, DYRK3, CDK13, PTPRD, FLT1, PTPRF, TRPM7, PTPRN2, PRKCI, PTPRR, PTPRT, HGF, MAPK10, STK3, NEK11, GAK, PPM1E, KSR2, PSEN1, RPS6KA2, GSK3B, RIPK1, CIT, MYLK, MELK, KALRN |
|  | 3.23 | Molecular Function FAT | metallopeptidase activity | 12 | 1.5E-07 | MMP20, ECE1, ADAMTS19, THSD4, NLN, MMP16, LTA4H, ADAMTS12, MIPEP, ADAM12, CPB2, ADAMTS2 |
|  |  | Biological Process FAT | proteolysis | 14 | 7.0E-03 | ADAMTS19, MMP16, MIPEP, HGF, UBE2L3, MMP20, ECE1, PSEN1, NLN, LTA4H, ADAMTS12, ADAM12, CPB2, ADAMTS2 |
